# Flexible electrochemical energy storage devices and related applications: recent progress and challenges

**DOI:** 10.1039/d4sc02139h

**Published:** 2024-06-28

**Authors:** Bo-Hao Xiao, Kang Xiao, Jian-Xi Li, Can-Fei Xiao, Shunsheng Cao, Zhao-Qing Liu

**Affiliations:** a School of Chemistry and Chemical Engineering/Institute of Clean Energy and Materials/Key Laboratory for Clean Energy and Materials, Guangzhou University Guangzhou 510006 China kxiao@gzhu.edu.cn lzqgzu@gzhu.edu.cn; b School of Materials Science & Engineering, Jiangsu University Zhenjiang 212013 China sscao@ujs.edu.cn

## Abstract

Given the escalating demand for wearable electronics, there is an urgent need to explore cost-effective and environmentally friendly flexible energy storage devices with exceptional electrochemical properties. However, the existing types of flexible energy storage devices encounter challenges in effectively integrating mechanical and electrochemical performances. This review is intended to provide strategies for the design of components in flexible energy storage devices (electrode materials, gel electrolytes, and separators) with the aim of developing energy storage systems with excellent performance and deformability. Firstly, a concise overview is provided on the structural characteristics and properties of carbon-based materials and conductive polymer materials utilized in flexible energy storage devices. Secondly, the fabrication process and strategies for optimizing their structures are summarized. Subsequently, a comprehensive review is presented regarding the applications of carbon-based materials and conductive polymer materials in various fields of flexible energy storage, such as supercapacitors, lithium-ion batteries, and zinc-ion batteries. Finally, the challenges and future directions for next-generation flexible energy storage systems are proposed.

## Abstract 2

This paper reviews advancements in flexible carbon-based and polymer gel materials for various types of energy storage systems, providing guidance for future development of next-generation wearable electronics.

## Graphical abstract

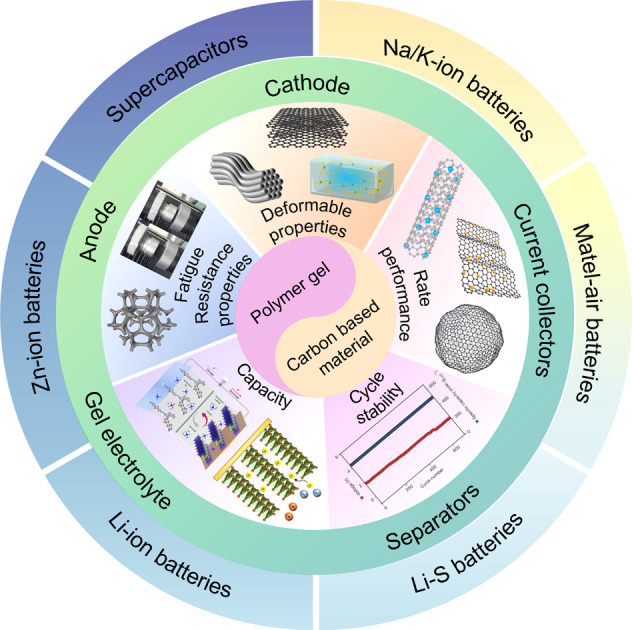

## 1. Introduction

The rapid consumption of fossil fuels in the world has led to the emission of greenhouse gases, environmental pollution, and energy shortage.^1,2^ It is widely acknowledged that sustainable clean energy is an effective way to solve these problems, and the use of clean energy is also extremely important to ensure sustainable development on a global scale.^3–5^ Over the past 30 years, great progress has been made in the development of electrochemical energy storage (EES) devices, such as rechargeable lithium-ion batteries (LIBs) and supercapacitors (SCs), which have been used in portable devices, electric vehicles, and stationary energy storage systems.^6–8^ With the increasing demand for energy storage devices, research on flexible electronic devices with flexible, folding and stretching functions is imminent, such as wearable electronic devices, electronic paper, smart clothing, electronic skins, displays, flexible smartphones, implantable medical devices, *etc.*^9–11^ To power these devices, it is necessary to develop flexible, scalable energy storage systems that can withstand mechanical deformation while maintaining their electrochemical properties.^12,13^ In general, LIBs and SCs are composed of several main components such as a positive electrode, negative electrode, isolation layer, electrolyte, fluid collector and packaging material.^14^ Due to the limitations of molding technology and traditional structure, the commercial LIBs and SCs currently used have high hardness and weight.^15–18^ Conventional electrodes are prepared by coating an electrode slurry (including active materials, conductive materials, and polymer adhesives) on a collector, then drying and pressing tightly.^19,20^ As a result, electrode materials easily fall off from the collector. As for the metal collectors, it is difficult for them to return to the initial state after repeated deformation, which leads to the deterioration of energy storage performance.^21^ In addition, the delaminated electrode material may penetrate the isolation layer, leading to short circuits and thermal runaway.^22^ Additionally, conventional liquid electrolytes inevitably exhibit leakage under deformation, thus necessitating the utilization of solid/quasi-solid gel electrolytes possessing superior mechanical properties and adhesion as an optimal solution to this issue.^23^

**Fig. 1:**
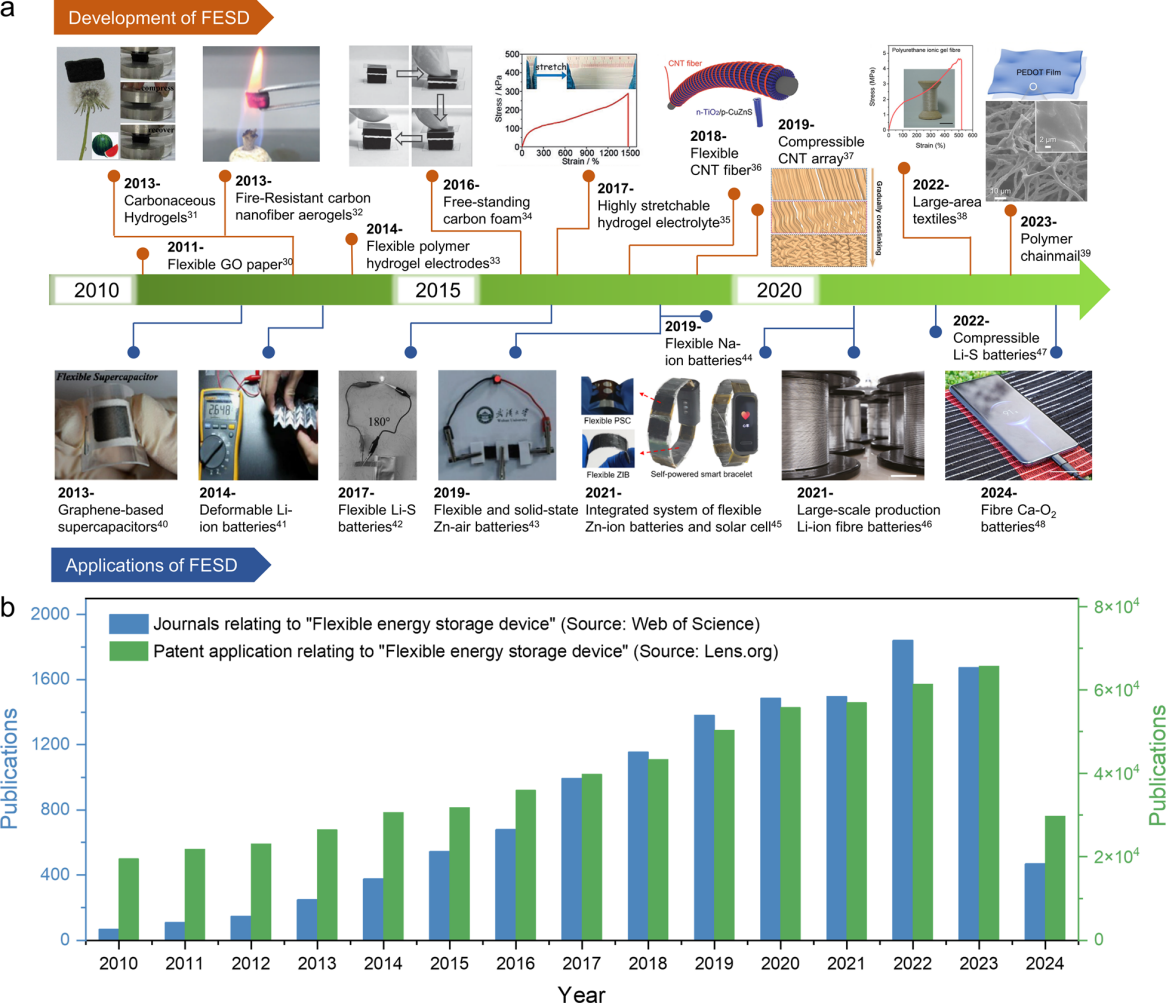
(a) Timeline showing the key development of flexible energy storage devices and their applications in wearable electronics.^30–48^ Reproduced with permission. (b) Summary of the publication records pertaining to “flexible energy storage device” in the Web of Science and Lens.org databases, with a search date of June 2024.

For flexible and scalable LIBs and SCs, all components are inevitably subjected to repeated elastic deformation, but traditional components do not have this advantage, so all components in LIBs and SCs should be selected to withstand continuous deformation.^24,25^ Among these components, the electrodes and electrolytes play a pivotal role in determining the mechanical and electrochemical properties of the system. Previous research has predominantly focused on investigating these two crucial elements.^26–29^Fig. 1a presents a comprehensive timeline illustrating the evolution and development of deformable electrodes and electrolytes for energy storage devices, as well as their applications in wearable electronics.^30–48^ The timeline categorizes these advancements based on material composition and functionality, showcasing the diverse progress achieved using carbon-based materials and organo-gels for developing wearable energy storage devices. The increasing number of published papers and patent applications (Fig. 1b) reflects that flexible energy storage has gradually attracted extensive attention from academic and industrial circles in recent years. These technological advancements are poised to revolutionize wearable electronics by enhancing their comfort, durability, and multifunctionality, ultimately paving the way for their commercialization. However, it is still in the early stages of research. Therefore, the exploration of novel material and structural design solutions for flexible and scalable EES remains an urgent and thorny challenge.

**Fig. 2:**
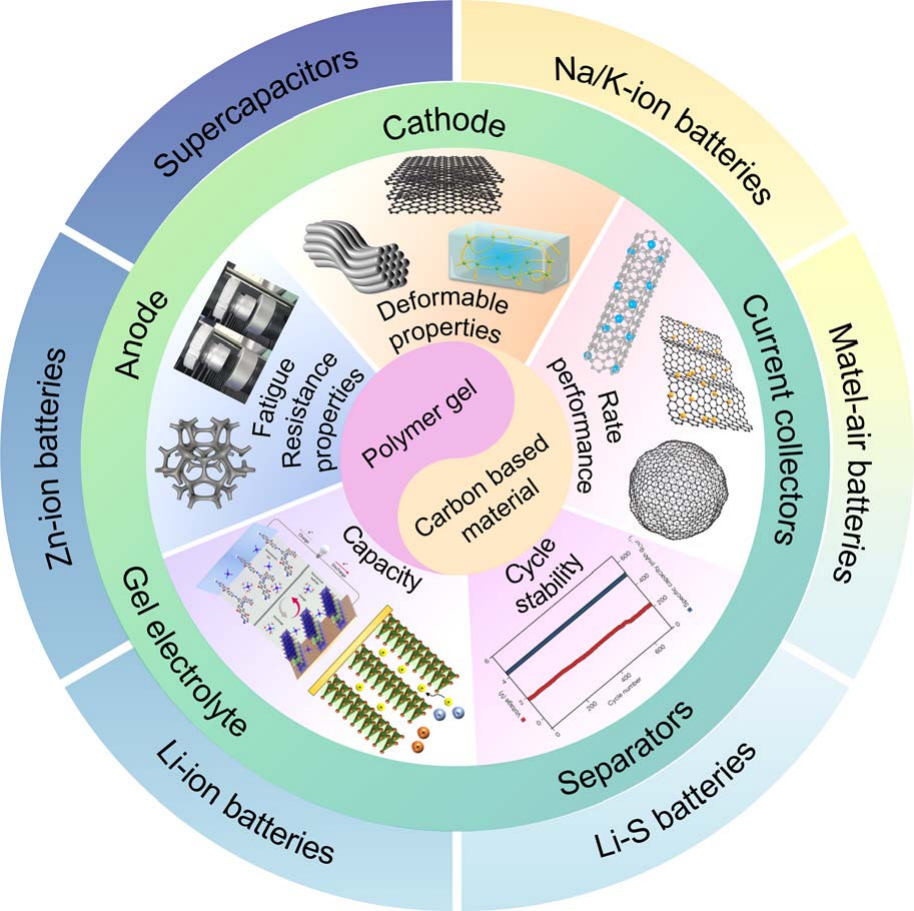
The outline map of this review.

In this review, we review the design, synthesis strategies, and recent advances of electrode and electrolyte materials for various flexible energy storage devices (Fig. 2). The review begins with a detailed discussion of synthetic strategies for flexible electrode materials and gel electrolytes in Section 2. Subsequent sections provide a comprehensive discourse on electrochemical energy storage systems currently employed in wearable electronics: SCs in Section 3, zinc-ion batteries (ZIBs) in Section 4, metal–air batteries in Section 5 within an aqueous system, lithium-ion batteries in Section 6, lithium–sulfur batteries (LSBs) in Section 7, and sodium ion batteries (SIBs) as well as potassium ion batteries (PIBs) in Section 8 within the organic system. Finally, we provide a comprehensive overview of strategies aimed at optimizing flexible electrode and electrolyte materials, as well as integrating flexible energy storage devices, to expedite the realization of next-generation flexible electronic devices.

## 2. Material design for flexible electrochemical energy storage devices

In general, the electrodes and electrolytes of an energy storage device determine its overall performance, including mechanical properties (such as maximum tensile/compressive strain, bending angle, recovery ability, and fatigue resistance) and electrochemical properties (including capacity, rate performance, and long-term cycling stability). To cater to the requirements of flexible electronic devices, it is imperative for electrodes and electrolytes to retain their original mechanical and electrochemical properties across diverse deformation states.

### 2.1 Flexible electrodes

There are two primary approaches to the design of flexible electrode materials: one involves transforming non-flexible materials into flexible ones through structural engineering, while the other entails combining active electrode materials with flexible substrates. Both strategies require further development to effectively address the increasing demand for wearable electronics.

#### 2.1.1 Structural engineering

**Fig. 3:**
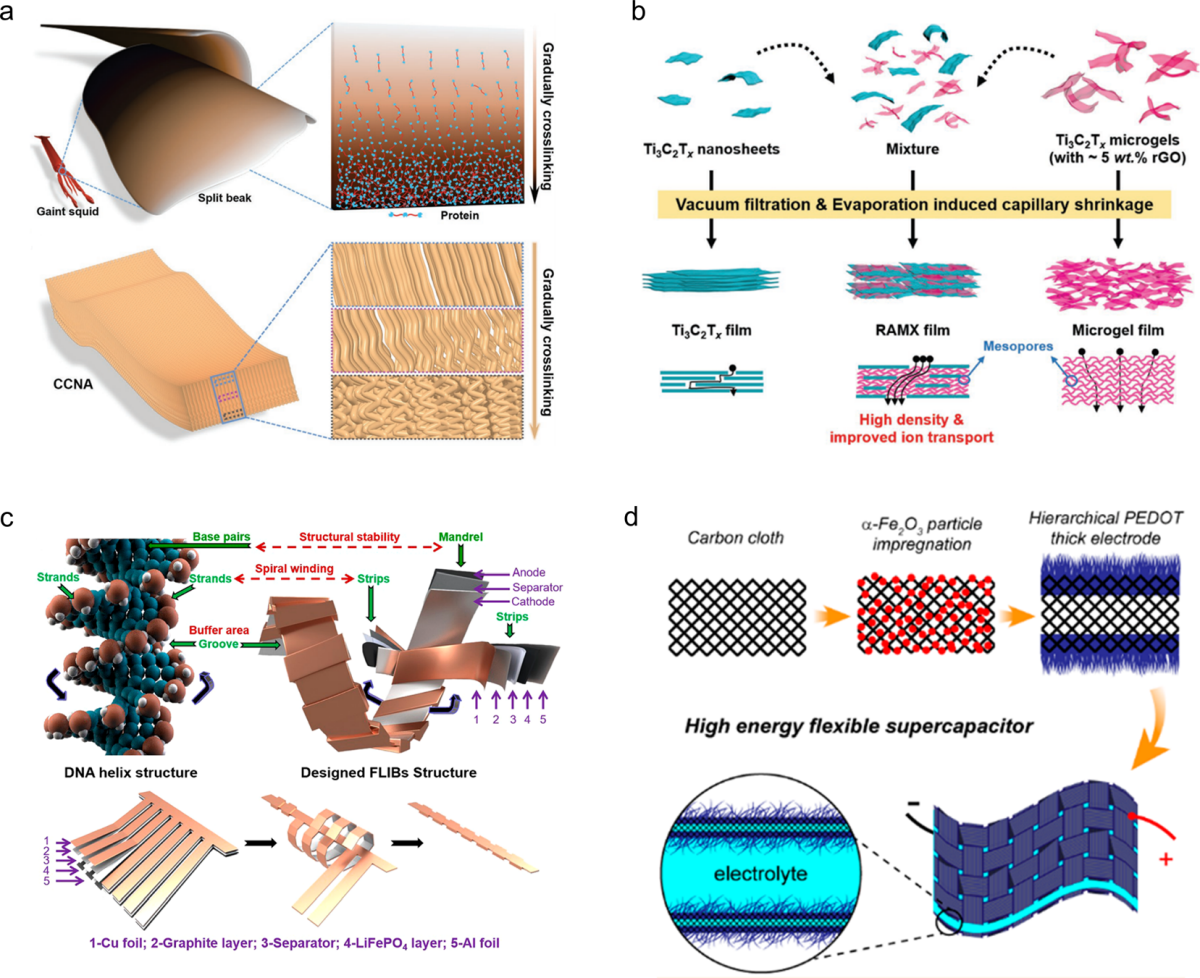
(a) Schematic illustration of a biomimetic freestanding carbon nanotube array designed to replicate the gradually crosslinked structure found in the beak of a giant squid.^37^ Reproduced with permission from ref. 37. Copyright 2019, Wiley-VCH GmbH. (b) Schematic representation of the preparation of Ti_3_C_2_T_*x*_ films.^49^ Reproduced with permission from ref. 49. Copyright 2021, Wiley-VCH GmbH. (c) Schematic representation of a helix-inspired battery designed with reference to the DNA structure.^50^ Reproduced with permission from ref. 50. Copyright 2022, American Chemical Society. (d) Schematic representation of *in situ* synthesis of polymer PEDOT on carbon cloth.^51^ Reproduced with permission from ref. 51. Copyright 2021, American Chemical Society.

The flexibility of the electrode material can be achieved through macroscopic or microscopic material structure design, in accordance with the specific requirements. For example, Peng *et al.*^37^ fabricated a carbon nanotube array with a gradient structure resembling the beak of a giant squid through vapor deposition, resulting in a progressively cross-linked architecture. This ingenious structural design enables the carbon nanotube arrays to offer reversible compressive strain up to 60%, exceptional fatigue resistance (100 000 cycles at 20% strain), and remarkable electrochemical stability across varying strains (Fig. 3a). The recombination strategy proposed by Tao *et al.*^49^ enables the construction of independent and flexible MXene thin film electrodes. The Ti_3_C_2_T_*x*_ microgels, isolated from 3D structured hydrogels, are reassembled with individual Ti_3_C_2_T_*x*_ nanosheets in varying mass ratios to form a densely packed 3D network in microscale, and a film morphology in macroscale. The resulting material demonstrates excellent bending properties (180°) and exhibits a high capacity of 736 F cm^−3^ at an ultrahigh scan rate of 2.0 V s^−1^ (Fig. 3b). Yin *et al.*^50^ developed a flexible LIB that draws inspiration from the helical structure of DNA. The battery architecture primarily consists of multiple rigid, thick energy stacks and some flexible, thin connectors. The thick energy stacks serve as the main storage units for energy, while the thin connectors are responsible for accommodating deformation. This design allows for stress buffering in the spiral strain region within the cell slot. As a result, this battery exhibits satisfactory capacity retention and stable energy output even under static, dynamic, and harsh *in situ* stress loads (Fig. 3c).

#### 2.1.2 Combination with elastomers

Traditional pseudocapacitive energy storage cathode materials such as MnO_2_,^52^ V_2_O_5_,^53^ and conductive polymers^54^ exhibit satisfactory theoretical specific capacity. However, when they are applied to flexible energy storage devices, the fabrication of flexible composites necessitates the use of other highly conductive substrates through hydrothermal, electrodeposition, and chemical vapor deposition methods. A typically substrate is carbon-based materials including carbon cloth,^55^ carbon fibers,^56^ carbon aerogel,^57^ and carbon foam^58^ which are commonly employed as flexible conductive substrates. And the optimization of the preparation process is crucial to prevent shedding of the active material grown on the substrate when it is deformed. For example, Wang *et al.*^51^ demonstrated a chemical strategy for controlling the oxidative radical polymerization process of conductive polymer PEDOT on carbon cloth using Fe_2_O_3_ particles as oxidant precursors. The reasonable spatial distribution of oxidants results in minimal increase in mechanical stiffness of the composite electrode material, which exhibits excellent adhesion even at higher mass loading. As a result, the PEDOT/CC composite electrode material displays high area capacitance (6.21 F cm^−2^) and maintains 94% of its initial performance under 180° bending (Fig. 3d). Wei *et al.*^59^ engineered and fabricated self-supporting hybrid nanostructures comprising ultra-long manganese dioxide nanowires integrated with graphene nanosheets as cathode materials for flexible ZIBs. This unique one-dimensional (1D)/two-dimensional (2D) nanostructure not only exhibits electrochemical synergy but also demonstrates exceptional mechanical flexibility, retaining its performance even under bending deformation. The composite demonstrates a specific capacity of 317 mA h g^−1^ at 0.1 A g^−1^, thereby retaining its original properties even in the folded state.

### 2.2 Flexible hydrogel electrolytes

Another crucial element of energy storage devices is the electrolyte, comprising inorganic salts and solvents with high conductivity. Within an electrolyte, the conductive salt undergoes dissociation into charge-carrying ions and shuttles between the positive and negative electrodes to facilitate charge transport. The electrochemical properties, such as conductivity, mobility rate, and diffusion coefficient, are determined by factors including ion concentration within the electrolyte, solvent type (inorganic/organic), and electrode interactions.

**Fig. 4:**
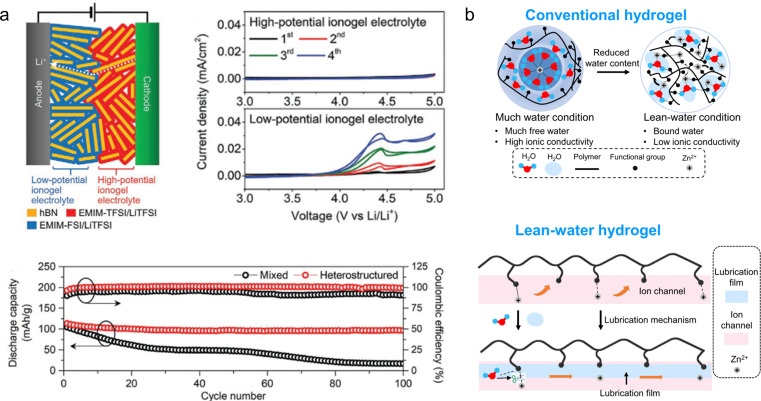
(a) Schematic of a solid-state lithium-ion battery using the layered heterostructure ionic gel electrolyte with two different ionic liquids and hexagonal boron nitride nanoplatelets and the electrochemical properties.^60^ Reproduced with permission from ref. 60. Copyright 2021, Wiley-VCH GmbH. (b) Schematic illustration of the ionic transportation mechanism of hydrogels.^61^

Gel electrolytes have been extensively utilized in the field of flexible energy storage due to their excellent mechanical stability and conductivity. To develop electrolytes suitable for flexible energy storage devices, it is imperative to modify the physical state of the electrolyte to a solid or quasi-solid form, thereby preventing any leakage during mechanical deformation. The commonly employed raw materials for gel preparation mainly include polyvinyl alcohol, polyacrylamide, polyethylene glycol, and polyacrylic acid. Gel electrolytes can be categorized into ionic gel electrolytes, hydrogel electrolytes, and organic gel electrolytes. For instance, C. Hersam *et al.*^60^ have reported on the development of an ion-gel electrolyte based on layered heterostructures utilizing two imidazole ionic liquids. This unique layered architecture exhibits a significantly expanded electrochemical window while maintaining exceptional ionic conductivity. In comparison to conventional ion-gel electrolytes, the heterogeneous nature of this layered ion-gel electrolyte enhances both the cycling stability and mechanical properties of LIBs (Fig. 4a). To solve the problems of low conductivity of solid gel electrolyte and poor interfacial stability of the Zn anode, Zhi *et al.*^61^ developed a water-deficient hydrogel electrolyte as an intermediate layer. The polymer backbone of the hydrogel features a polymeric zwitterion, with sulfonate end groups that exhibit both hydrophilic and zincophilic properties, while the coordination unit is provided by the zinc salt. This unique structure enables impressive ion conductivity (2.6 × 10^3^ S cm^−1^) even under water-poor conditions, demonstrating excellent long-term cycling stability (91% at 5C for 4000 cycles), as well as strong adhesion and strain tolerance (Fig. 4b).

## 3. Flexible supercapacitors

SCs represent a highly promising candidate for flexible/wearable energy storage devices owing to their high power density, long cycle life and fast charge/discharge rates.^62^ Categorized based on the energy storage mechanism, they can be classified into electrical double layer capacitors and pseudo-capacitors.^63^ Electrical double layer capacitors store charge through the electrostatic adsorption process that occurs at the interface between the electrode and electrolyte, while pseudo-capacitors store charge through electron gain/loss *via* a rapid and reversible redox reaction on the surface of the electrode. Although the flexible electrode materials and hydrogel electrolytes investigated thus far exhibit commendable mechanical properties, there remains space for improvement in the electrochemical performance of these devices. The electrode materials for flexible SCs are classified and discussed in this section, encompassing three categories: compressible electrodes, foldable electrodes, and stretchable electrodes. Furthermore, a concise overview is provided on the electrolytes and current collectors employed in flexible SCs.

### 3.1 Flexible electrodes

#### 3.1.1 Compressible electrodes

Freestanding three-dimensional (3D) carbon-based materials, such as carbon aerogels and carbon foams, exhibit great potential as advanced materials for compressible SCs.^64^ These carbon-based electrode materials possess a highly porous structure and rough surface, resulting in their super-hydrophilicity and low densities. Moreover, their regular honeycomb internal architecture enables efficient stress distribution along the vertical axis to the lateral directions, thereby preventing fracture of the carbon framework and facilitating elastic energy storage.^65^

**Fig. 5:**
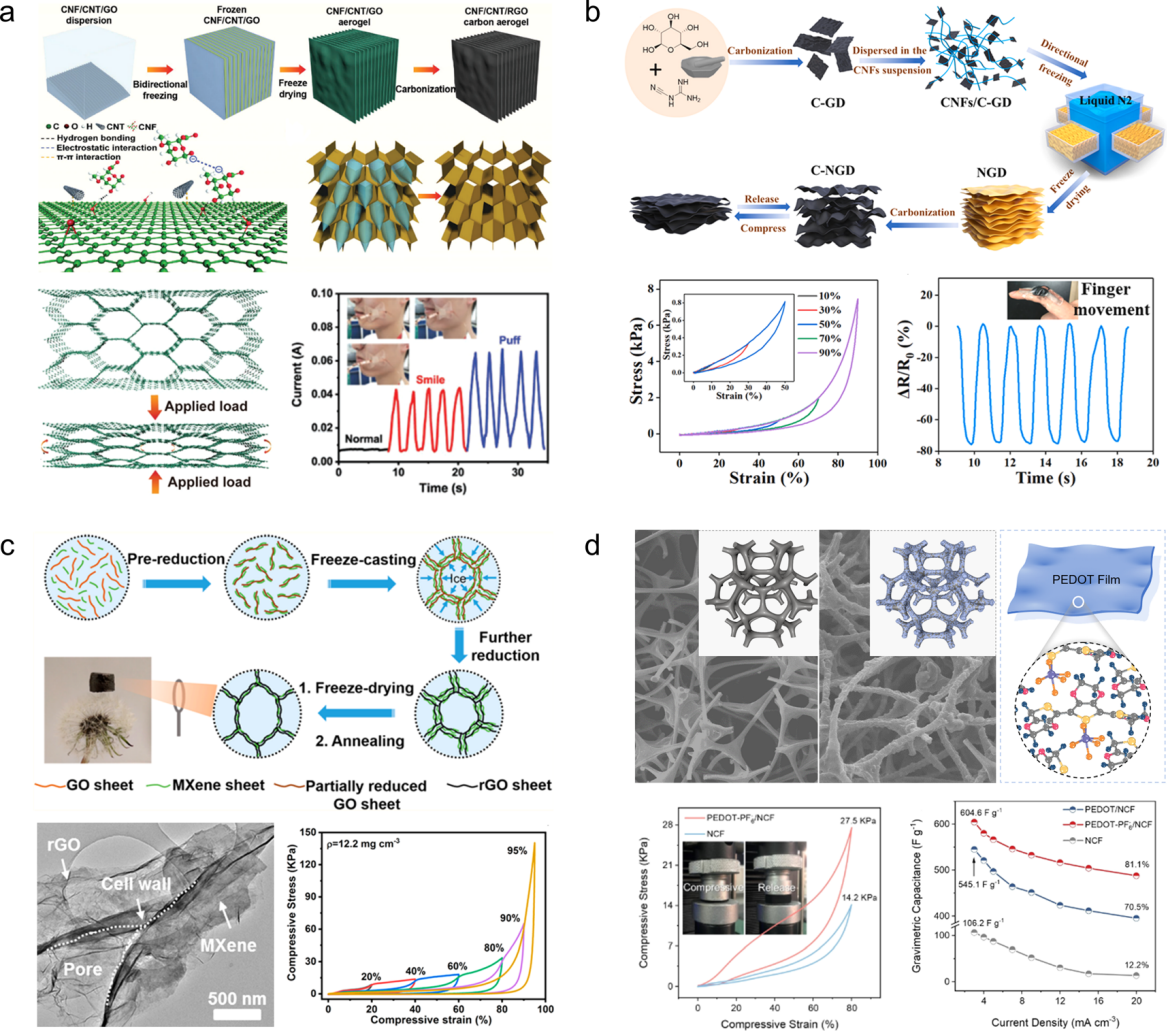
(a) Schematic diagram for the preparation of CNF/CNT/rGO carbon aerogels and their practical applications.^66^ Reproduced with permission from ref. 66. Copyright 2022, Wiley-VCH GmbH. (b) Preparation process of C-NGD carbon aerogels and their mechanical and electrochemical properties.^57^ Reproduced with permission from ref. 57. Copyright 2021, Elsevier. (c) Schematic diagram and digital photographs of the fabrication process for the MXene/rGO; SEM image and galvanostatic charge–discharge curves of MXene/rGO.^67^ Reproduced with permission from ref. 67. Copyright 2021, American Chemical Society. (d) Schematic illustration of the synthesis process of PEDOT-PF-6, along with the mechanical and electrochemical properties of PEDOT-PF-6.^39^ Reproduced with permission from ref. 39. Copyright 2023, Wiley-VCH GmbH.

Zhang and his colleagues^66^ prepared cellulose nanofibrils/carbon nanotubes/reduced graphene oxide (CNF/CNTs/rGO) composite carbon aerogels through bi-directional freezing and annealing treatments. By introducing cellulose nanofibers and carbon nanotubes into the reduced graphene oxide, a regular layered porous structure was formed, effectively inhibiting the stacking of graphene sheets. Therefore, the CNF/CNTs/rGO exhibited excellent performance with a capability to undergo 10 000 cycles at 50% compressive strain. Furthermore, it demonstrated outstanding area capacitance (109.4 mF cm^−2^ at 0.4 mA cm^−2^) as well as long-term cycling stability (88% retention after 5000 cycles at 50% compression strain). The CNF/CNTs/rGO can be used as a strain sensor to capture biological signals from the human body (Fig. 5a). Ye *et al.*^57^ synthesized nitrogen-doped carbon aerogels (C-NGDs) through a biomass-mediated approach, utilizing cost-effective glucose and dicyandiamide as raw materials instead of the expensive graphene oxide and carbon nanotubes. Owing to the highly stable wave-layered structure of the aerogels, it can withstand up to 95% compressive strain. Moreover, when assembled into a symmetric device, it exhibits an impressive specific capacity of 220.2 F g^−1^ at a current density of 0.5 A g^−1^, thus establishing itself as a versatile material for applications in biosensing, flexible electronics, and energy storage and conversion devices (Fig. 5b). However, despite the 3D carbon aerogel's potential as a SC electrode material, it cannot meet the required capacity demands, hence the need for introducing pseudocapacitive materials to enhance their electrochemical performance. M. Razal *et al.*^67^ developed a super-elastic and highly loaded MXene/rGO composite aerogel through reduction, freeze-drying and annealing processes. The highly loaded MXene was attached to the graphene wall to form a robust cellular scaffold (Fig. 5c). Due to the synergistic effect of the pseudocapacitive material MXene and graphene, the composite aerogel exhibits not only excellent mechanical stability (60% strain for 1000 cycles) but also high specific capacity (397 F g^−1^ at 0.5 A g^−1^). Similarly, Liu and his colleagues^39^ proposed a “polymer chainmail” strategy, wherein electrochemically stabilized anion-doped PEDOT is synthesized on a 3D nitrogen-doped carbon foam skeleton through chemical *in situ* polymerization (Fig. 5d). Due to the film-forming characteristics during the PEDOT polymerization process and excellent electrochemical performances, the resulting composites exhibit a synergistic enhancement of mechanical properties (1000 cycles of compression at 80% strain) and electrochemical properties (604.6 F g^−1^ at 3 mA cm^−3^).

#### 3.1.2 Foldable electrodes

**Fig. 6:**
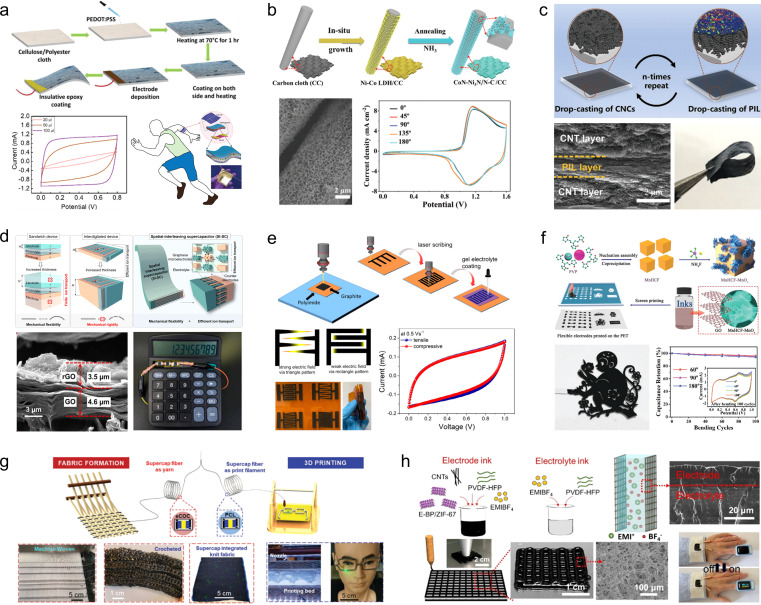
(a) Scheme for the fabrication of PEDOT:PSS-based electrodes on cloth.^68^ (b) Schematic diagram of the synthesis process for CoN–Ni_3_N/N–C/CC.^69^ Reproduced with permission from ref. 69. Copyright 2021, Wiley-VCH GmbH. (c) Schematic illustration of the preparation process and digital photographs of (CNC/PIL)-*n*L.^70^ Reproduced with permission from ref. 70. Copyright 2022, Wiley-VCH GmbH. (d) Spatial-interleaving SCs based on graphene microelectrodes.^71^ Reproduced with permission from ref. 71. Copyright 2022, American Chemical Society. (e) Fabrication of interdigitated carbon-based electrodes.^72^ (f) Schematic representation of the synthesis steps for MnHCF–MnO_*x*_, the photograph of large-scale printed patterned electrodes, and characterization of their electrochemical properties.^73^ Reproduced with permission from ref. 73. Copyright 2020, Wiley-VCH GmbH. (g) Schematic diagram of machine-weaving and 3D printing from thermally drawn supercapacitor fibers.^74^ Reproduced with permission from ref. 74. Copyright 2020, Wiley-VCH GmbH. (h) Illustration of the 3D printing of all-integrated solid-state flexible supercapacitors.^75^ Reproduced with permission from ref. 75. Copyright 2021, Wiley-VCH GmbH.

Commercially available carbon and cellulose cloths with low cost, high conductivity, high toughness, and excellent foldability are promising substrates for flexible electrode materials. Various types of energy storage active materials can be easily synthesized on carbon or cellulose cloth by chemical redox, vapor phase deposition and electrodeposition to enhance capacity and energy density. Dahiya *et al.*^68^ fabricated a textile based wearable SC using sweat as an electrolyte by coating PEDOT:PSS as an electroactive material onto a cellulose cloth substrate (Fig. 6a). The conjugated polymer forms an electrochemical double layer on the surface of electrodes upon interaction with authentic sweat, and its performance exhibits significant dependence on both the quantity and concentration of sweat. Consequently, this developed SC holds promising potential for applications in human perspiration sensing. Zhao and his coworkers^69^ used a mild solvothermal method followed by post-nitriding treatment to synthesize honeycomb-shaped CoN–Ni_3_N/N–C nanosheets *in situ* on a flexible carbon cloth. The problem of structural transition metal nitrides during electrochemical reactions was successfully addressed by employing the strategy of growing transition metal nitrides on a conducting substrate (Fig. 6b). The rational design of the hierarchically integrated nanostructures yields a substantial specific capacity (1.48 F cm^−2^ at 0.5 mA cm^−2^) and an extended cycle life (93.3% capacity retention after 10 000 cycles), while maintaining proper functionality across diverse bending angles.

In addition to commercially available fabrics, a wide range of homemade flexible 2D thin-film carbon-based electrode materials have been extensively developed for application in foldable SCs. These materials can be tailored with diverse internal structures based on specific requirements using techniques such as laser cutting, repeated drop-casting, and 3D printing, thereby overcoming the limitations imposed by the original structure of commercial flexible substrates. Pan *et al.*^70^ prepared CNC/PEDOT:PSS flexible films with hierarchical structure by alternating deposition of carbon nanocoils and PEDOT:PSS (Fig. 6c). The interconnected CNCs within the porous structure serve as high-speed channels for ion transport, while the presence of the PEDOT:PSS conductive film ensures structural stability and enhances interlayer electron transfer kinetics. Consequently, these thin-film electrodes exhibit excellent bendability and electrochemical properties, achieving an area capacitance of 1402.5 mF cm^−2^ at 0.25 mA cm^−2^. Moreover, when assembled into solid-state SCs based on CNC/PEDOT:PSS, they demonstrate a wide potential window of 2.0 V and maintain initial performances even under various bending angles. Li and his coworkers^71^ developed a spatially interleaved SC using the laser cutting method (Fig. 6d). In this approach, graphene microelectrodes are reversely stacked layer by layer in 3D space, with narrow gaps strategically incorporated to enhance ion transport within the electrolyte. The unique interlaced structure of graphene microelectrodes imparts excellent mechanical flexibility to the spatially interleaved SCs, which exhibits negligible capacity decay even after undergoing 10 000 bending tests. Moreover, it achieves an impressive area-ratio capacitance of 36.46 mF cm^−2^ within a compact thickness of only 100 μm, thereby showcasing its potential for seamless integration into wearable electronic devices. Lee *et al.*^72^ improved the process of laser engraving to design flexible SCs, wherein the electrode layer was designed with intersecting sharp edges to induce a pronounced electric field at the corners of the electrode fingers, thereby augmenting charge accumulation near the electrode surface (Fig. 6e). Wu *et al.*^73^ employed screen-printing technology to fabricate electrode materials composed of manganese hexacyanoferrate–manganese oxide and electrochemically reduced graphene oxide composites (MnHCF–MnO_*x*_/ErGO), which can be tailored into diverse patterns as required (Fig. 6f). At a current density of 1.0 A g^−1^, a high specific capacity of 467 F g^−1^ was achieved, and the capacitance remained stable for 100 cycles under different bending angles (60°, 90°, and 180°).

In recent years, the widespread utilization of 3D printing technology in the domain of flexible energy storage devices has been attributed to its capability to design electrode materials or energy storage devices with diverse geometries based on specific requirements. This addresses the issues related to limited scalability, flexibility, and adaptability encountered by flexible energy storage devices. Fink *et al.*^74^ pioneered the fabrication of fiber supercapacitors through a top-down approach, wherein macroscopic prefabricated components were thermally stretched into 100 meter-long energy storage fibers possessing excellent mechanical strength and moisture resistance (Fig. 6g). Wu *et al.*^75^ developed a heterogeneous nanostructured black phosphorus/metal–organic framework hybrid (E-BP/ZIF-67), which exhibited high specific surface area and remarkable electrochemical activity (Fig. 6h). The highly active nanohybrid along with the poly(vinylidene difluoride–hexafluoropropylene) binder functioned as stabilizing ink constituents, enabling successful construction of this electrode material using 3D printing technology in a fully integrated manner. The resulting solid-state flexible supercapacitor demonstrated an ultra-high volumetric energy density of 109.8 mW h cm^−3^, long-term stability over 12 000 cycles, and exceptional deformable performance.

#### 3.1.3 Stretchable electrodes

**Fig. 7:**
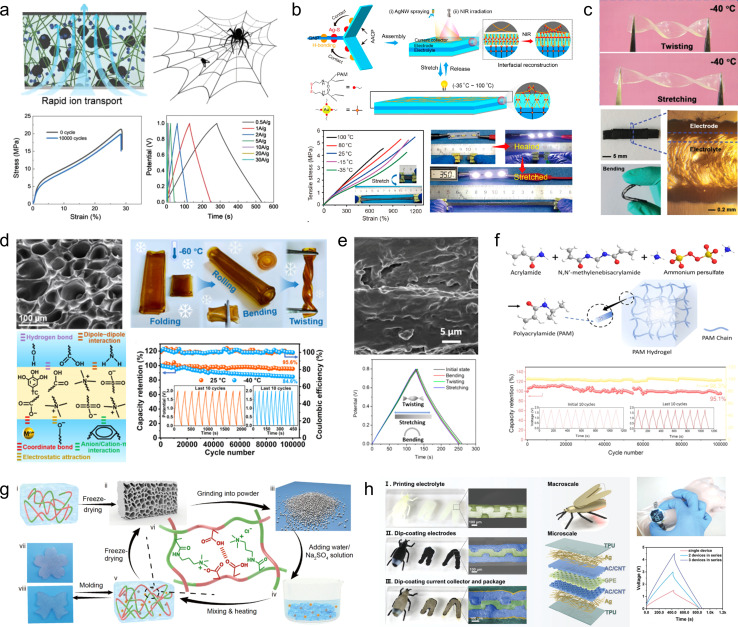
(a) Electrodes based on the preparation of 3D structural adhesives inspired by spider webs.^78^ Reproduced with permission from ref. 78. Copyright 2021, Wiley-VCH GmbH. (b) Schematic illustrations of the preparation of the AACP hydrogel electrode.^79^ Reproduced with permission from ref. 79. Copyright 2022, American Chemical Society. (c) Exhibition of twisting and stretching for hydrogel webs.^80^ Reproduced with permission from ref. 80. Copyright 2021, Wiley-VCH GmbH. (d) Anti-freezing and self-adhesive polyzwitterionic hydrogel electrolyte.^81^ Reproduced with permission from ref. 81. Copyright 2021, American Chemical Society. (e) Morphology and electrochemical performance of the MMT/PVA membrane.^82^ Reproduced with permission from ref. 82. Copyright 2020, American Chemical Society. (f) Illustration of the synthesis of the polyacrylamide (PAM) hydrogel and its cycle stability.^83^ Reproduced with permission from ref. 83. Copyright 2020, Wiley-VCH GmbH. (g) Fabrication scheme for the regenerated hydrogel electrolyte.^84^ Reproduced with permission from ref. 84. Copyright 2021, Springer Nature. (h) Optical photographs, structural schematics, and electrochemical properties of double-layer supercapacitors assembled based on gel-like polymer electrolytes.^85^ Reproduced with permission from ref. 85. Copyright 2023, Wiley-VCH GmbH.

The unique stretchable mechanical properties, excellent electrical conductivity, and energy storage properties of polymer hydrogels make them widely employed in the design and fabrication of electrode materials for stretchable SCs.^76,77^ Yu and his colleagues,^78^ referencing the structure of a spider's web, synthesized an adhesive by the biosynthesis process, and used it to attach the electroactive material to the interconnected and entangled bacterial cellulose 3D structure of the web to obtain stretchable electrodes (Fig. 7a). The electrode was able to withstand a high tensile strength of 19.5 MPa (tensile strain of 29%). In addition, thanks to the spiderweb-type 3D binder, the electrode material possesses a high mass loading and thus exhibits a high specific capacitance of 225.8 F g^−1^ at 0.5 A g^−1^. Yu *et al.*^79^ synthesized a silver nanowire (AgNW)/silver nanoparticle-decorated carbon nanotube (Ag–CNT)/polyacrylamide (PAM) hydrogel (AACP) through *in situ* polymerization of polyacrylamide on the AgNW/Ag–CNT, which effectively preserved the interconnection between the networks of Ag–CNT and AgNW after applying a thin layer of PAM (Fig. 7b). Consequently, the resulting hydrogel exhibited an impressive electrical conductivity of 965 S cm^−1^. Benefiting from the structural advantages, the composites effectively dissipate crack energy through shape deformation of the honeycomb network, formation of crack bridges in the intertwined network, and utilization of Ag–SR bond-induced crack sharing in the integrated structure. Additionally, hydrogels exhibit an impressive tensile strain of 2430%. SCs assembled using an AACP electrode and multifunctional GNP electrolytes demonstrate exceptional temperature adaptability (−35–80 °C) as well as remarkable cycling stability (97.1% capacity retention after 10 000 cycles at −35 °C).

### 3.2 Flexible electrolytes

Hydrogel electrolytes have attracted a great deal of attention in flexible and safe SCs due to the risk of electrolyte leakage from SC devices under deformation or bending. However, the interfacial contact problem between the hydrogel electrolyte and electrodes as well as the environmental instability are the key factors restricting the development of hydrogel supercapacitors. Therefore, in recent years, people have been committed to developing new high-performance hydrogel electrolytes. Gao *et al.*^80^ developed a nucleotide-tackified adhesive organo-hydrogel electrolyte (P(AM-*co*-DMAEMA)/gelatine) that facilitates intimate electrode-hydrogel electrolyte contact while reducing the interfacial contact resistance (Fig. 7c). Moreover, the hydrogel exhibited excellent mechanical properties and freezing resistance, ensuring the absence of delamination and displacement between the electrolyte and electrodes even under severe deformation conditions. Additionally, these SCs operated reliably for 5000 cycles at temperatures as low as −20 °C. However, further reduction in temperature leads to a significant decline in both the conductivity and mechanical properties of these SCs, thereby severely compromising interfacial adhesion. To address this issue, Yang and his colleagues^81^ developed an anti-freezing and self-adhesive polyzwitterionic hydrogel electrolyte (PZHE) using an autocatalytic nano-enhancement strategy (Fig. 7d). The PZHE maintained good interfacial adhesion even at a low temperature of −60 °C. Additionally, the ZnCl_2_-filled salt-in-water type PZHE facilitated ion migration channels and enhanced the reversibility of the Zn metal electrodes, effectively reducing side reactions and prolonging cycle life. As a result, the Zn^2+^ ion hybrid capacitor achieved a high energy density of 80.5 W h kg^−1^ with excellent cycle stability of up to 100 000 cycles. On the other hand, to address the issue of instability exhibited by conventional hydrogel electrolytes under high temperature, Chen *et al.*^82^ developed a montmorillonite/polyvinyl alcohol (MMT/PVA) hydrogel electrolyte, which exhibited enhanced thermal stability due to the presence of montmorillonite and a reduced freezing point by incorporating dimethyl sulfoxide into the solution (Fig. 7e). As a result, the electrolyte demonstrated high conductivity ranging from 0.17 × 10^−4^ to 0.76 × 10^−4^ S cm^−1^ over a wide temperature range (−50 °C to 90 °C). Moreover, the assembled devices exhibited excellent capacity performance and mechanical flexibility. In addition to its exceptional temperature adaptability, it is crucial not to overlook the influence of flexible gel electrolyte modification on the cycling stability of devices. Chen *et al.*^83^ have successfully developed a mechanically flexible water-in-salt hydrogel electrolyte based on a chloride ion-facilitated de-solvation mechanism within hydrated ZnCl^+^–(H_2_O)_*n*−1_ (with *n* = 1–6) clusters (Fig. 7f). This innovative approach not only enhances the energy storage capacity of porous carbon materials but also improves the reversibility of Zn metal electrodes. The resulting zinc-ion hybrid capacitor exhibits an impressive energy density of 217 W h kg^−1^ and demonstrates outstanding cycle life with up to 100 000 cycles.

To accommodate supercapacitor electrode materials with customizable external geometries obtained through 3D printing preparation, studies on gel electrolytes prepared using 3D printing technology have also been reported. Hydrogel electrolytes, due to their excellent mechanical strength, reproducibility, and plasticity, can serve as a suitable substrate for 3D printing. For instance, Wang *et al.*^84^ developed a robust hydrogel by random copolymerization of 3-(methacryloylamino)-propyl trimethylammonium chloride and methacrylic acid, utilizing various interactions such as hydrophilic hydrogen bonding and polyanionic interactions along with entanglement of hydrophobic polymer chains (Fig. 7g). The resulting hydrogel exhibits mechanical stability and self-repairing properties owing to the interplay between the hydrogen bonding network and polyanions/cations. When employed in flexible supercapacitors, this electrolyte can also function as a binder that integrates active materials into the polymer network while significantly reducing interfacial resistance between the electrolyte and electrodes. Consequently, the assembled device maintains stable mechanical and electrochemical properties even after undergoing over 100 consecutive bending cycles. Zhou *et al.*^85^ proposed an innovative fabrication method for flexible supercapacitors wherein a gel electrolyte template was initially prepared *via* 3D printing followed by assembly of electrodes, collectors, and encapsulation materials through the dip-coating technique, thus enabling realization of flexible supercapacitors possessing both an internal three-dimensional structure and overall 3D geometry that can be easily integrated into diverse electronic products (Fig. 7h).

## 4. Flexible zinc-ion batteries

Compared to LIBs employing organic electrolytes, aqueous ZIBs have garnered attention due to their superior safety profile, cost-effectiveness, and simplified assembly process (eliminating the need for glovebox operation).^86^ Since the Zn anode enables two-electron participation in the electrochemical reaction, it exhibits a remarkable specific capacity of 820 mA h g^−1^ alongside a low redox potential of −0.76 V (*vs.* the standard hydrogen electrode).^87^ Therefore, ZIBs hold great promise as highly efficient energy storage devices for next-generation wearable electronics.^88^

### 4.1 Foldable electrodes

Manganese-based or vanadium-based oxides, such as V_2_O_5_, VO_2_, MnO_2_, and Mn_3_O_4_, are commonly employed as cathode materials for aqueous ZIBs.^89,90^ However, the limited electrical conductivity inherent in these metal oxides poses challenges to their rate performance and cycling stability. To address this issue, these active materials are usually compounded with various carbon-based materials, such as carbon nanotubes, reduced graphene oxide and carbon cloth.^91,92^

**Fig. 8:**
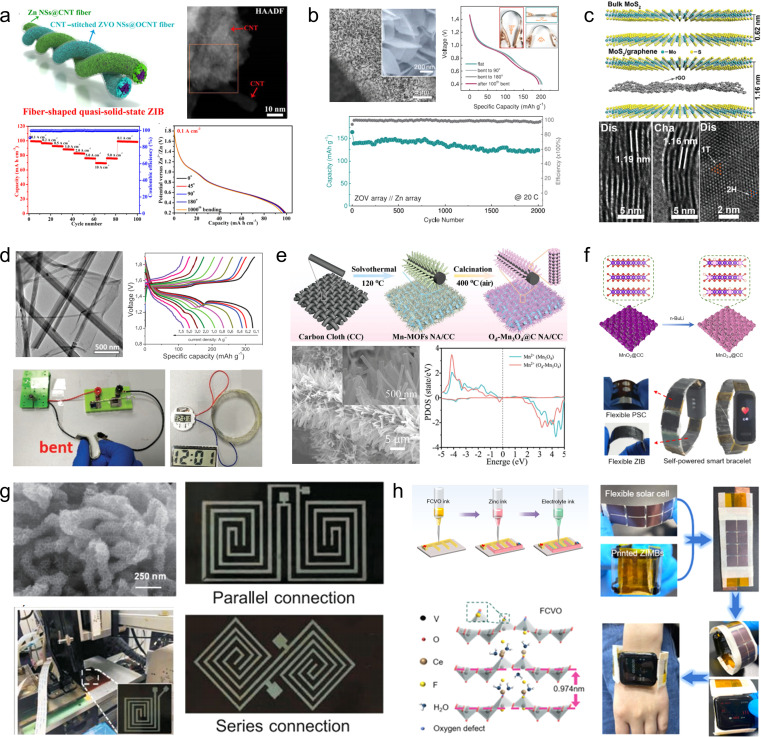
(a) Schematic illustration of the fiber-shaped quasi-solid-state Zn//ZVO ZIB and its electrochemical properties.^93^ Reproduced with permission from ref. 93. Copyright 2020, American Chemical Society. (b) Morphology and electrochemical properties of a ZVO array cathode.^94^ Reproduced with permission from ref. 94. Copyright 2018, Wiley-VCH GmbH. (c) Crystal structures of MoS_2_/graphene and its phase transition.^95^ Reproduced with permission from ref. 95. Copyright 2021, Wiley-VCH GmbH. (d) Morphology and electrochemical properties of the MnO_2_/rGO nanocomposite.^59^ Reproduced with permission from ref. 59. Copyright 2020, Wiley-VCH GmbH. (e) Illustration of the synthesis procedures of the Od-Mn_3_O_4_@C NA/CC nanostructure.^96^ Reproduced with permission from ref. 96. Copyright 2020, Wiley-VCH GmbH. (f) Schematic fabrication process of the MnO_2−*x*_@CC and its practical applications.^45^ Reproduced with permission from ref. 45. Copyright 2021, American Chemical Society. (g) Photograph of the multinozzle printing system and a heating plate of the 3D printing equipment printer.^97^ (h) Schematic of the fabrication process and optical photographs of the flexible zinc ion micro-batteries through 3D printing.^98^

1D carbon nanotubes can serve as linking materials for transition metal oxides, thereby addressing the issue of mechanical instability in the direction perpendicular to 2D nanosheets. For instance, Wang *et al.*^93^ developed a strategy involving the use of 1D carbon nanotubes stitched with Zn_3_(OH)_2_V_2_O_7_·2H_2_O (CNT-stitched ZVO) 2D nanosheets directly grown on oxidized carbon nanotube fibers (Fig. 8a). The incorporation of stitched carbon nanotubes onto the ZVO nanosheets not only imparted electrical conductivity but also enhanced mechanical properties. Consequently, even when subjected to bending at 180°, the composites exhibited no active material detachment and retained their original properties. Moreover, assembling a full cell with Zn NSs@CNT fibers (carbon nanotube fibers electrodeposited with zinc nanosheets) as anodes demonstrated exceptional performance stability over extended cycling periods (69.7% capacity retention after a 100-fold increase in current density and 88.6% capacity retention after 2000 cycles).

2D graphene nanosheets are commonly used as flexible and conductive scaffolds for layered transition metal oxides. For instance, Fan *et al.*^94^ fabricated layered Zn orthovanadate and Zn nanosheets on a 3D porous graphene substrate to serve as the cathode and anode, respectively, in flexible ZIBs (Fig. 8b). Due to the ordered structure of the active material, the full cell assembled with quasi-solid hydrogel electrolyte exhibited exceptional rate capability at 50C and demonstrated excellent cycling stability (2000 cycles at a rate of 20C), effectively suppressing dendrite growth. Furthermore, the fully functional cell displayed remarkable mechanical flexibility within a bending angle range of 0 to 180°. Li *et al.*^95^ innovatively embedded graphene nanosheets into MoS_2_ channels (Fig. 8c). The sandwich structured MoS_2_/graphene nanosheets promoted the diffusion of Zn^2+^ as well as the penetration of the electrolyte solution, along with strong structural stability. The composite thus demonstrated high-rate performance (285.4 mA h g^−1^ at 0.05 A g^−1^ with 141.6 mA h g^−1^ at 5 A g^−1^) and long-term cycling stability (88.2% capacity retention after 1800 cycles). Through a series of *ex situ* characterizations, the authors validated that the introduction of graphene nanosheets enabled highly reversible phase transition between 2H-MoS_2_ and 1T-MoS_2_, thus elucidating the energy storage mechanism. Similarly, Wei *et al.*^59^ fabricated a composite material comprising of ultra-long manganese dioxide nanowires and graphene nanosheets as a flexible cathode for ZIBs (Fig. 8d). Benefiting from the strong electrochemical synergy provided by the unique 1D/2D nanostructures, the MnO_2_/rGO composite demonstrates an enhanced specific capacity (317 mA h g^−1^) in its conventional state and maintains normal operation even when folded at 180°.

Commercial carbon cloths are also commonly used to prepare cathode materials for flexible ZIBs. Wang *et al.*^96^ synthesized oxygen-deficient Mn_3_O_4_@C nanorod arrays (Od-Mn_3_O_4_@C NA/CC) on carbon cloth by carbonizing the manganese metal–organic framework (Fig. 8e). In addition to enhancing the conductivity of Mn_3_O_4_, they successfully improved the stability of the MnO_6_ octahedral structure, effectively inhibiting Mn^2+^ dissolution. As a result, the assembled full cell achieved an impressive capacity retention of 84.1 mA h g^−1^ (up to 95.7% of the initial capacity) after 12 000 cycles at 5 A g^−1^. Similarly, Tan *et al.*^45^ fabricated a cathode material for ZIBs by growing defective MnO_2−*x*_ nanosheets on carbon cloth (Fig. 8f). A Li treatment during the preparation process was employed to expand the layer spacing of MnO_2_ and induce oxygen vacancies. Compared to pristine MnO_2_/CC, the utilization capacity, rate capability, and cycling stability of MnO_2−*x*_/CC as a cathode were significantly enhanced. Even at an ultra-high mass loading of 25.5 mg cm^−2^, the specific capacity reached 3.63 mA h cm^−2^. Self-powered wristbands based on flexible ZIBs assembled with MnO_2−*x*_/CC and integrated with solar cells demonstrated their potential in powering commercial wearable electronic devices.

The 3D printing technology has been utilized for the fabrication of carbon-based cathode materials in flexible ZIBs. For instance, Ma *et al.*^97^ developed a bendable electrode material by employing the 3D printing technique to prepare manganese dioxide-coated carbon nanotube (CNT@MnO_2_) ink. The resulting Zn//CNT@MnO_2_ flexible ZIBs exhibited a stable capacity of 63 μA h cm^−2^ at 0.4 mA cm^−2^, with only a maximum capacity loss of 2.72% when subjected to bending in different states (Fig. 8g). Tan *et al.*^98^ reported an F/Ce co-doped V_2_O_5_ printing ink that demonstrated improved specific capacity, rate performance, and long-term cycling stability due to increased layer spacing and introduction of oxygen defects in V_2_O_5_ after co-doping (Fig. 8h). The electrodes fabricated using the 3D printing technology displayed high-quality loading and excellent mechanical properties, leading to ultra-high capacity (10.1 mA h cm^−2^) and energy density (8.1 mW h cm^−2^) in assembled flexible ZIBs.

The primary obstacle currently impeding the advancement of ZIBs lies in the uncontrolled growth of dendrites on the anode Zn electrode, which is primarily attributed to excessive local current density and electric field imbalance during Zn deposition. This flaw is inevitably exacerbated under conditions of device bending and deformation. Consequently, it is imperative to consider the issue of dendrite growth when designing flexible anode materials for ZIBs.

**Fig. 9:**
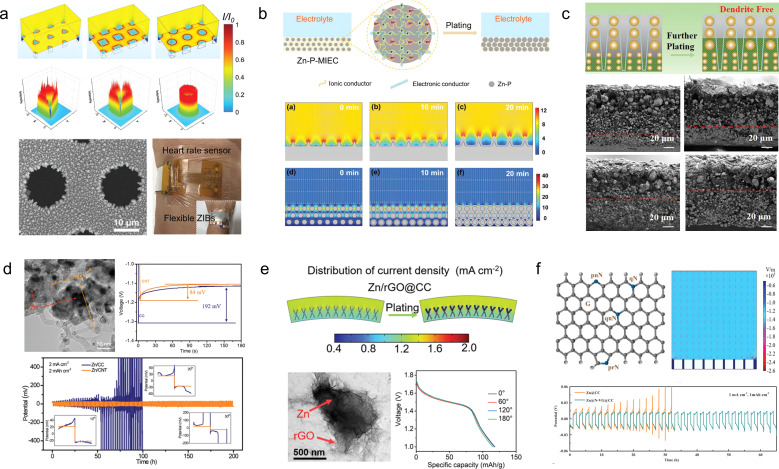
(a) Theoretical simulations of current density redistributions and morphology of the biomimetic honeycomb anode.^99^ Reproduced with permission from ref. 99. Copyright 2023, Wiley-VCH GmbH. (b) Schematic illustrations and electric field simulation on the mixed ionic–electronic conductor scaffold for Zn-powder.^100^ Reproduced with permission from ref. 100. Copyright 2022, Wiley-VCH GmbH. (c) Schematic diagram of desirable bottom-up and dendrite-free Zn deposition behavior in the Zn powder-based anode with gradient particle size.^101^ Reproduced with permission from ref. 101. Copyright 2023, Wiley-VCH GmbH. (d) TEM images and electrochemical performances of the Zn/CNT sample.^102^ Reproduced with permission from ref. 102. Copyright 2019, Wiley-VCH GmbH. (e) Electric field simulation, TEM image, and electrochemical performances of Zn/rGO@CC electrodes.^103^ Reproduced with permission from ref. 103. Copyright 2023, Wiley-VCH GmbH. (f) Schematic diagram and models of the electric field distributions for the Zn@N-VG@CC electrode.^104^ Reproduced with permission from ref. 104. Copyright 2021, Wiley-VCH GmbH.

Generally, there are two design concepts for flexible ZIB anodes. The first involves employing structural engineering techniques to make the Zn anode flexible. For example, Duan *et al.*^99^ have developed a topology-optimized bionic honeycomb Zn anode that effectively enhances the distribution of current, stress, and thermal fields within the anode (Fig. 9a). This innovative structure achieves a multi-field modulation effect, leading to improved electrochemical and mechanical stability. Notably, the electrode exhibits an impressive cycle life of 2000 h (at a current density of 30 mA cm^−2^) and demonstrates exceptional rate performance at 20C. Furthermore, this anode stands out for its remarkable performance while maintaining cost-effectiveness.

Flexible Zn anode materials can also be prepared using Zn powder as a raw material by the tape-casting technology. Liang *et al.*^100^ mixed Zn powder, ethylene vinyl acetate copolymer and carbon nanotubes sufficiently and then cast them onto a film, which was peeled off from the mold after drying to obtain a Zn-P-MIEC film (Fig. 9b). This freestanding flexible electrode material consists of a hybrid ionic–electronic conductor scaffold formed by carbon nanotubes and ethylene vinyl acetate copolymer, providing flexibility to the material while protecting the Zn powder from corrosion in aqueous solutions. The resulting zinc anode enables homogenization of the Zn^2+^ flux during repetitive galvanizing/stripping processes for stable and reversible cycling of Zn^2+^. Remarkably, even after 1270 h of cycling, only a minimal increase in voltage hysteresis was observed in batteries assembled with Zn-P-MIEC. Guan *et al.*^101^ employed a similar method to fabricate thin-film anode materials based on Zn powder, but they introduced a novel concept of gradient particle size (Fig. 9c). By reducing the particle size of the Zn powder and electrode porosity with increasing depth, a vertical gradient particle flux is generated, facilitating bottom-up Zn deposition and exfoliation while impeding dendrite growth. Consequently, these prepared electrodes exhibit exceptional cycling stability even under high current density and capacity conditions (5 mA cm^−2^, 5 mA h cm^−2^), achieving a cycle life of up to 130 h.

A more streamlined and efficient approach to mitigating dendrite growth in ZIBs involves the construction of a flexible 3D conductive framework, which serves as a galvanized/stripped support. Lu *et al.*^102^ utilized 3D carbon nanotubes as a flexible substrate and a galvanized support, resulting in a notable enhancement in the coulombic efficiency of the Zn/CNTs compared to the pristine deposited Zn electrode (Fig. 9d). This improvement can be attributed to the preparation of Zn/CNTs with reduced Zn nucleation overpotential and a more uniform distribution of electric field. Moreover, it exhibited excellent stability, operating at 2 mA cm^−2^ for 200 h without any observed dendrite growth. Lu *et al.*^103^ fabricated a Zn/3D reduced graphene oxide composite on carbon cloth substrates *via* one-step co-electrodeposition (Fig. 9e). The incorporation of a 3D reduced graphene oxide network not only enhanced the electrical conductivity and wettability of the electrode materials but also resulted in a more uniform electric field distribution and lower local current density, effectively suppressing the growth of zinc dendrites. The electrode material exhibited excellent long-term cycling stability for 1000 h at 1 mA cm^−2^, while the assembled full battery with MnO_2_ demonstrated remarkable rate performance and maintained its original properties even under bending conditions. Further optimization was conducted by Guan and his colleagues,^104^ resulting in the proposal of an *in situ* growth method for 3D nitrogen-doped vertical graphene nanosheets on carbon cloth (Fig. 9f). This approach also enables the realization of flexible Zn anodes without dendrite growth. The introduction of nitrogen-containing groups into the 3D graphene nanosheets significantly enhances the interaction between Zn^2+^ and the carbon substrate, thereby reducing the overpotential for Zn nucleation and facilitating uniform distribution of Zn nuclei on the surface of the anode.

### 4.2 Stretchable electrodes

**Fig. 10:**
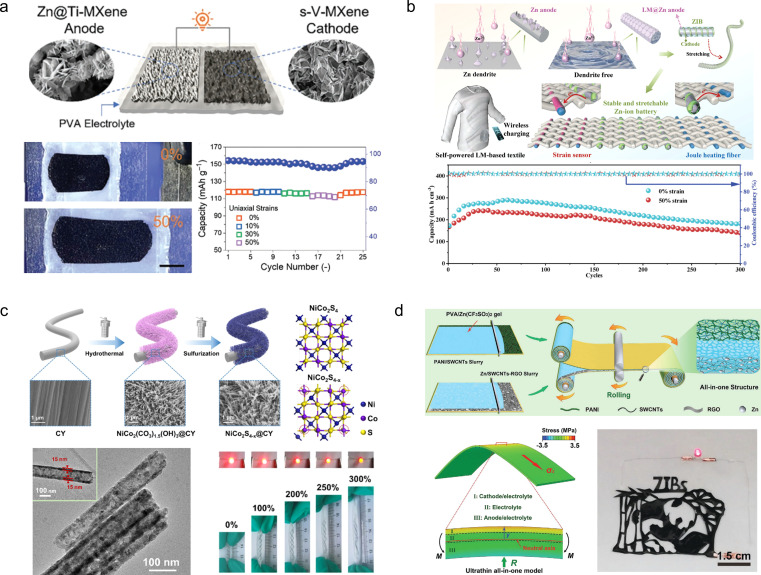
(a) Schematic illustration (left) and digital photographs at different deformation stages (right) of a stretchable Zn^2+^ ion hybrid battery.^105^ Reproduced with permission from ref. 105. Copyright 2021, Wiley-VCH GmbH. (b) Scheme of a self-powered LM-based electronic textile integrating a ZIB with a strain sensor, a Joule heating fiber, and a wireless charging part.^106^ Reproduced with permission from ref. 106. Copyright 2023, Wiley-VCH GmbH. (c) The fabrication schematic diagram of NiCo_2_S_4−*x*_@CY.^107^ Reproduced with permission from ref. 107. Copyright 2023, Springer Nature. (d) The design of ultrathin all-in-one ZIBs.^108^ Reproduced with permission from ref. 108. Copyright 2021, Wiley-VCH GmbH.

Compared to foldable batteries, stretchable batteries play an equally crucial role in wearable electronic devices. Chen *et al.*^105^ designed a stretchable ZIB which is based on a V_2_CT_*x*_ cathode and a Zn-modified Ti_3_C_2_T_*x*_ film composition (Fig. 10a). During electrode fabrication, both MXene-based electrodes were intentionally designed with a wrinkle-like micro-texture, enabling reversible folding/unfolding behaviour upon stretching to effectively mitigate in-plane stress. Consequently, the assembled device exhibits remarkable tensile strain resistance up to 50% while experiencing only minimal capacity attenuation (from 118.5 to 103.6 mA h g^−1^) under tensile conditions. However, the tensile properties of MXene films are not inherent, thereby limiting the maximum tensile strain of this electrode material. Guan and his colleagues^106^ have reported an intrinsically stretchable liquid metal fiber anode (LM@Zn) (Fig. 10b). The inherent stretchability and liquid properties of LM@Zn ensure that the fiber ZIBs possess excellent tensile properties. When subjected to a tensile strain of 50%, the fiber ZIBs based on the LM@Zn anode retain 83% of their initial capacity. Furthermore, owing to its remarkable zincophilic and alloying effect, the LM@Zn anode exhibits a more uniform Zn deposition process along with enhanced Zn (002) crystal orientation. Consequently, the assembled ZIB demonstrates exceptional cycling stability, enabling continuous operation for over 800 h. Wei *et al.*^107^ prepared NiCo_2_S_4−*x*_ arrays with tunable sulfur vacancies grown on carbon yarn (NiCo_2_S_4−*x*_@CY) through a simple hydrothermal method, utilizing carbon yarns as substrate materials that possess intrinsic stretchable properties (Fig. 10c). The introduction of sulfur vacancies was found to promote the surface remodelling and phase transition of NiCo_2_S_4−*x*_, leading to enhanced conductivity and charge storage kinetics. As a cathode material for ZIBs, the composite exhibited favourable discharge capacity (271.7 mA h g^−1^) and excellent cycling performance (70.9% capacity retention at 5 A g^−1^). Moreover, when integrated into stretchable ZIBs as the base material, NiCo_2_S_4−*x*_@CY demonstrated exceptional tensile stability and durability under various mechanical deformations. However, during the process of the device being stretched, the individual components within it may separate from each other, thus affecting the overall performance of the cell. In order to solve this problem, Niu *et al.*^108^ successfully developed a stretchable ultrathin all-in-one ZIB through a blade coating and rolling assembly process, wherein the PANI/SWCNTs cathode, Zn/SWCNTs anode, and PVA/Zn(CF_3_SO_3_)_2_ gel electrolyte were seamlessly integrated into a single unit (Fig. 10d). This innovative integrated structure effectively prevents any relative displacement or detachment between adjacent components, ensuring continuous and efficient ion and/or load transfer capability even under external deformation while maintaining exceptional structural and electrochemical stability. Moreover, this battery can be customized into various shapes and structures to meet specific requirements.

### 4.3 Flexible electrolytes

A solid gel electrolyte is required to replace the aqueous zinc salt solution in flexible ZIBs. The high-performance gel electrolyte not only enhances device deformability but also improves anti-freezing properties, inhibits anode Zn dendrite formation and side reactions to a certain extent, thereby enhancing cycling stability. Therefore, the development of flexible hydrogel electrolytes holds significant importance in assembling flexible ZIBs.

**Fig. 11:**
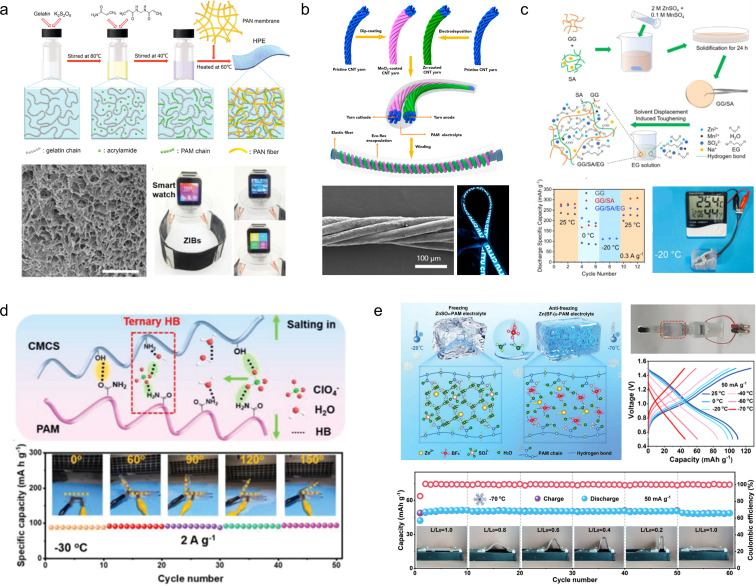
(a) Synthesis diagram, morphology and application of an HPE.^109^ (b) Schematic diagram of the fabrication and encapsulation of the yarn ZIBs.^110^ Reproduced with permission from ref. 110. Copyright 2018, American Chemical Society. (c) Schematic illustration of the fabrication of GG/SA/EG hydrogel electrolytes.^111^ Reproduced with permission from ref. 111. Copyright 2021, Elsevier. (d) Schematic diagram and performance of the CSAM-C hydrogel.^112^ Reproduced with permission from ref. 112. Copyright 2022, Wiley-VCH GmbH. (e) Schematic diagram and mechanism of the anti-freezing hydrogel electrolytes.^113^ Reproduced with permission from ref. 113. Copyright 2023, Wiley-VCH GmbH.

Zhi *et al.*^109^ developed a polyacrylamide-based hierarchical polymer electrolyte (HPE) by grafting polyacrylamide (PAM) onto gelatine chains embedded in a network of electrostatic spinning fiber membranes made from polyacrylonitrile (PAN), which enhanced the mechanical strength and conductivity of the polymer electrolyte (Fig. 11a). The assembled full cell with an MnO_2_/CNTs cathode demonstrated remarkable specific capacity (306 mA h g^−1^) and excellent cycling stability (97% capacity retention after 1000 cycles at 2772 mA g^−1^). Due to the exceptional mechanical stability of the layered hydrogel, the battery exhibits outstanding wear resistance and safety, enabling normal operation under extreme conditions such as cutting, bending, beating, *etc.* Subsequently, Zhi *et al.*^110^ have developed a high-performance, stretchable zinc-ion battery with a double-helical structure utilizing an HPE as a gel electrolyte and MnO_2_/CNTs as electrodes. The cell exhibits exceptional programmability and customizability while being able to withstand 300% tensile strain (Fig. 11b).

However, conventional hydrogel electrolytes exhibit a significant loss of flexibility at low temperatures and a notable decrease in conductivity, resulting in the attenuation of electrochemical properties. To address this issue, Zhou *et al.*^111^ developed GG/SA/EG hydrogels by combining two natural polymers, guar gum (GG) and sodium alginate (SA), which possess excellent antifreeze properties. The inclusion of ethylene glycol (EG) forms stable molecular clusters with H_2_O molecules and competes with hydrogen bonds in water, leading to a reduction in the saturated vapor pressure of water and consequently lowering the freezing point. Moreover, the GG/SA/EG hydrogels demonstrate remarkable mechanical properties with capacity retention rates of 87.2% and 78.3%, respectively, after undergoing 1000 bending cycles (Fig. 11c). Zhang *et al.*^114^ fabricated a highly flexible polysaccharide hydrogel incorporating a low concentration of Zn(ClO_4_)_2_ salt, wherein the ClO_4_^−^ anion, water, and polymer chains establish ternary and weak hydrogen bonds. This interaction enhances the mechanical properties of the polymer chains while disrupting the hydrogen bonding network among water molecules, leading to a significant reduction in electrolyte solidification point. Simultaneously, it diminishes free water content and effectively suppresses hydrogen precipitation side reactions as well as dendrite growth at the Zn anode (Fig. 11d). In order to achieve enhanced freezing resistance and mechanical properties, Niu *et al.*^113^ developed a hydrogel electrolyte (Zn(BF_4_)_2_–PAM) based on Zn(BF_4_)_2_ and PAM. The BF_4_^−^ anions interact with water molecules through strong O–H⋯F hydrogen bonds due to the highly electronegative nature of fluorine atoms, replacing the weak O–H⋯O hydrogen bonds between water molecules and effectively inhibiting ice crystal formation at low temperatures. As a result, the Zn(BF_4_)_2_–PAM hydrogel remains unfrozen even at −70 °C, enabling the construction of freeze-resistant flexible ZIBs that exhibit excellent cycling stability and consistent electrochemical performance under low temperature conditions as well as during bending (Fig. 11f).

## 5. Flexible metal–air batteries

The rapid advancement of portable and wearable electronic devices has led to widespread interest in flexible metal–air batteries due to their safe operation, low cost, high energy density, and eco-friendliness.^55^ However, the ORR and OER of the air electrode often experience sluggish kinetics under significant polarization conditions that rely heavily on efficient and stable catalysts.^115^ Additionally, conventional electrolytes face significant challenges in semi-open systems such as water evaporation at high temperatures or gel freezing at low temperatures leading to battery failure in harsh environments.^116^ To promote the development of flexible metal–air batteries, researchers have recently developed various new flexible air cathode catalysts and gel electrolytes, which are mainly used in Li–air batteries (LABs), Zn–air batteries (ZABs), and Al–air batteries (AABs).

### 5.1 Li–air batteries

Due to their reasonable theoretical energy density (∼400 W h kg^−1^) and energy efficiency (>90%), LABs have garnered significant attention in the realm of metal–air batteries.^117^ Despite the synthesis of air electrodes with porous structures and organic/aqueous electrolytes, conventional lithium–air batteries are manufactured with inflexible integral architectures that fail to meet the demands of wearable electronic devices. Furthermore, LABs still encounter substantial challenges such as side reactions, limited recyclability, and electrolyte leakage.

**Fig. 12:**
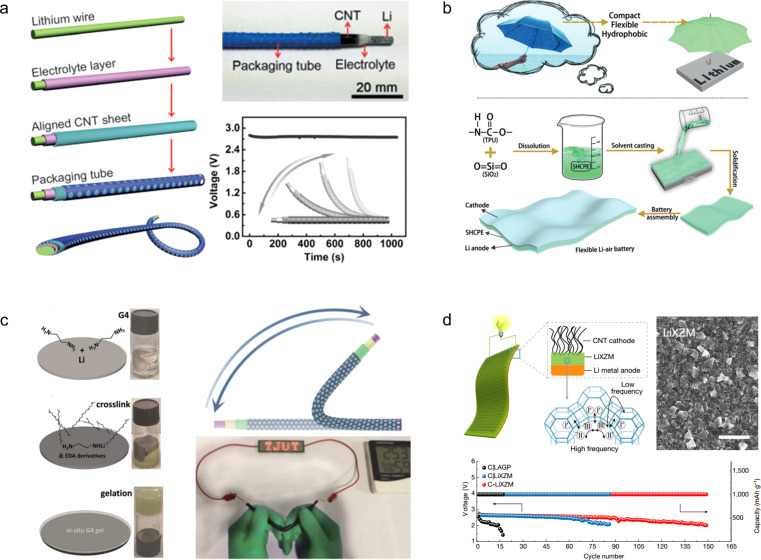
(a) Schematic fabrication of a fibrous lithium–air battery and its strain stability.^118^ Reproduced with permission from ref. 118. Copyright 2016, Wiley-VCH GmbH. (b) Schematic diagram illustrating the protection of the lithium anode and the fabrication process of a flexible LAB.^119^ Reproduced with permission from ref. 119. Copyright 2019, Wiley-VCH GmbH. (c) Schematic diagram illustrating the *in situ* preparation process of gel electrolyte and physical demonstration showcasing the normal operation of the flexible lithium–air battery assembled using this procedure in a bent state.^120^ Reproduced with permission from ref. 120. Copyright 2019, Wiley-VCH GmbH. (d) Schematic diagram of the Li^+^ ion conduction mechanism, morphology, and long-term cycling stability of Li^+^ ion exchange zeolites.^121^ Reproduced with permission from ref. 121. Copyright 2021, Springer Nature.

To develop an electrochemical energy storage system compatible with wearable electronics, Peng *et al.*^118^ fabricated a novel fibrous LAB featuring a coaxial structure that integrates an external carbon nanotube cathode and an internal Li wire anode within a polymer gel electrolyte, rendering it fibrous in nature. The incorporation of the gel electrolyte effectively hinders air diffusion onto the Li anode, thereby lowering its corrosion rate. Consequently, the resulting LAB exhibits excellent flexibility, braiding capability, and cycling stability (Fig. 12a).

To enhance the protection of the anode in lithium–air batteries, novel gel electrolytes with superior mechanical properties have been developed. For instance, Zhang *et al.*^119^ drew inspiration from the umbrella structure to develop a robust hydrophobic composite polymer electrolyte film that exhibits exceptional flexibility, hydrophobicity, and stability for safeguarding the lithium anode. The electrolyte is composed of thermoplastic polyurethane and hydrophobic silica nanoparticles, effectively preventing cell deformation and flooding. By mitigating the risks of puncture and heat-induced short-circuit fires, this innovation significantly enhances the safety profile of wearable LABs. Furthermore, the *in situ* preparation of the electrolyte film ensures its uniform distribution on the surface of the Li anode, thereby substantially retarding corrosion and dendrite growth while leveraging its inherent hydrophobic properties to prevent undesired side reactions (Fig. 12b). The tetraethylene glycol dimethyl ether gel electrolyte was prepared *in situ* on the lithium anode surface by Ding *et al.* through a crosslinking reaction.^120^ Notably, the inclusion of a polymer-free additive allows for the maintenance of high ionic conductivity within the gel, while the *in situ* preparation method ensures excellent interfacial contact between the electrodes. Thanks to this enhanced conductivity, the LABs assembled with this gel electrolyte exhibit stable operation for 1175 h in air (Fig. 12c).

The inherent mechanical stability of the organic gel electrolyte effectively safeguards the Li anode, but its low conductivity remains a limiting factor in its development. Despite efforts to enhance conductivity through the incorporation of inorganic electrolyte additives, they have not yet resolved the issue of unstable performance in air. To solve this problem, Yu *et al.*^121^ introduced a flexible solid zeolite electrolyte—a thin Li^+^ ion exchange zeolite membrane—through an innovative *in situ* assembly strategy that integrated it with both the Li anode and carbon nanotubes at the cathode (Fig. 12d). Due to the inherent chemical stability in air as well as high conductivity of zeolites, this all-solid-state LAB exhibited remarkable specific capacity (12 020 mA h g^−1^ for carbon nanotubes) and cycle stability (149 cycles at 500 mA g^−1^ and 1 mA h g^−1^).

### 5.2 Zinc–air batteries

Since Zn participates in a redox reaction involving the transfer of two electrons, the theoretical specific energy density of Zn–air batteries reaches 1086 W h kg^−1^, which surpasses that of LABs.^122^ Moreover, ZABs exhibit cost-effectiveness, safety, and environmental friendliness, positioning them as potential candidates for next-generation electrochemical energy storage and conversion systems.^123,124^ However, the sluggishness of the oxygen reduction reaction at the air cathode and the vulnerability of water electrolytes and Zn anodes to external conditions impede their further advancement.^125,126^ Therefore, developing affordable, high-performance, and stable cathode catalysts along with temperature-responsive gel electrolytes is essential for practical implementation of flexible ZABs in wearable electronic devices.^127^

#### 5.2.1 Flexible cathode catalysts

**Fig. 13:**
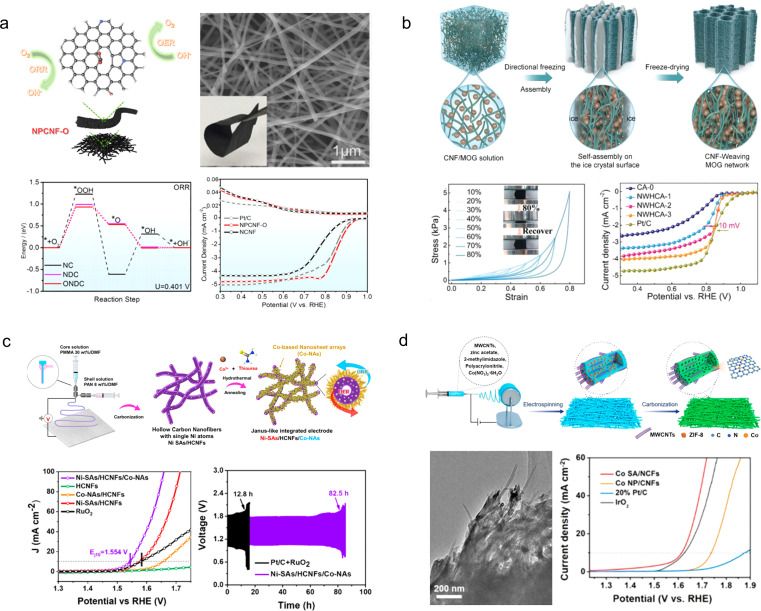
(a) Schematic illustration and electrochemical properties of the NPCNF–O catalyst.^129^ Reproduced with permission from ref. 129. Copyright 2022, American Chemical Society. (b) Schematic of the preparation procedure of the CNF-MOG by the nanofiber weaving strategy.^56^ Reproduced with permission from ref. 56. Copyright 2023, Wiley-VCH GmbH. (c) Illustration for the preparation of Ni-SAs/HCNFs/Co-NAs.^130^ Reproduced with permission from ref. 130. Copyright 2022, American Chemical Society. (d) Schematic illustration of the electrospinning process for the fabrication of Co SA/NCFs.^131^ Reproduced with permission from ref. 131. Copyright 2022, American Chemical Society.

Due to their inherent flexibility, carbon nanofibers can be used as independent air cathodes for the assembly of portable and flexible ZABs.^128^ For instance, Dong *et al.*^129^ designed a nitrogen and oxygen co-doped porous carbon nanofiber (NPCNF–O) by employing a simple electrospinning technique with β-cyclodextrin as a pore inducer and oxygen modifier (Fig. 13a). Through both DFT calculations and experimental investigations, it was confirmed that the carboxyl groups effectively modulated the local charge density of the nitrogen-doped carbon matrix, optimized the adsorption energy of intermediates, and reduced reaction energy barriers, thereby enhancing the catalytic activity towards oxygen. The NPCNF–O design exhibited exceptional activities in terms of ORR (*E*_1/2_ = 0.85 V *vs.* RHE) and OER (*E*_*J*=10_ = 1.556 V *vs.* RHE). Liu and his colleagues^56^ employed a nanofiber weaving technique to fabricate 3D ultra-light, ultra-elastic nanofiber woven hybrid carbon assemblies (NWHCA) by incorporating N and P co-doping into carbon aerogels (Fig. 13b). The resultant material not only demonstrated foldability but also exhibited remarkable compressibility, enduring up to 80% compressive strain while fully recovering its original shape.

Moreover, the compressive stress retained 94% of the initial stress even after undergoing 5000 cycles of compression and release. When used as an ORR electrocatalyst, NWHCA displayed exceptional catalytic activity with an onset potential of 0.93 V *vs.* RHE, a half-wave potential of 0.84 V *vs.* RHE, and outstanding electrochemical stability.

To further enhance the catalytic activity of the air cathode in flexible ZABs, cost-effective and high-performance ORR/OER catalysts can be integrated onto flexible porous carbon nanofibers. For instance, Liu *et al.*^130^ developed Ni single atoms coordinated with O and N for OER activity, as well as a Co_3_O_4_@Co_1−*x*_S nanosheet array for ORR activity, which were integrated on the inner and outer walls of flexible hollow carbon nanofibers (Ni-SAs/HCNFs/Co-NAs), respectively (Fig. 13c). As stand-alone bifunctional oxygen catalysts with flexibility, Ni-SAs/HCNFs/Co-NAs demonstrate remarkable ORR (*E*_1/2_ = 0.89 V *vs.* RHE) and OER (*E*_*J*=10_ = 1.554 V *vs.* RHE) activities, rapid electron/ion transfer kinetics, large electrochemically active specific surface area, and excellent mechanical flexibility. The assembled flexible all-solid-state ZABs exhibit outstanding cycling stability exceeding 80 h. Zhang *et al.*^131^ successfully fabricated N-doped porous carbon nanofibers interconnected by nanotubes, incorporating Co single atoms through electrospinning (Fig. 13d). The atomically dispersed Co species provide a plethora of catalytic sites for ORR/OER reactions with exceptional reactivity. The abundant pyridine N moieties not only serve as active sites but also effectively anchor the Co atoms, forming a stable Co–N_4_ configuration that enhances the activity and stability of the catalyst. Moreover, the internal CNTs enhance the flexibility and mechanical strength of the porous fibers. As an air cathode without binders, the prepared catalyst exhibits remarkable durability over 600 hours at a current density of 10 mA cm^−2^.

**Fig. 14:**
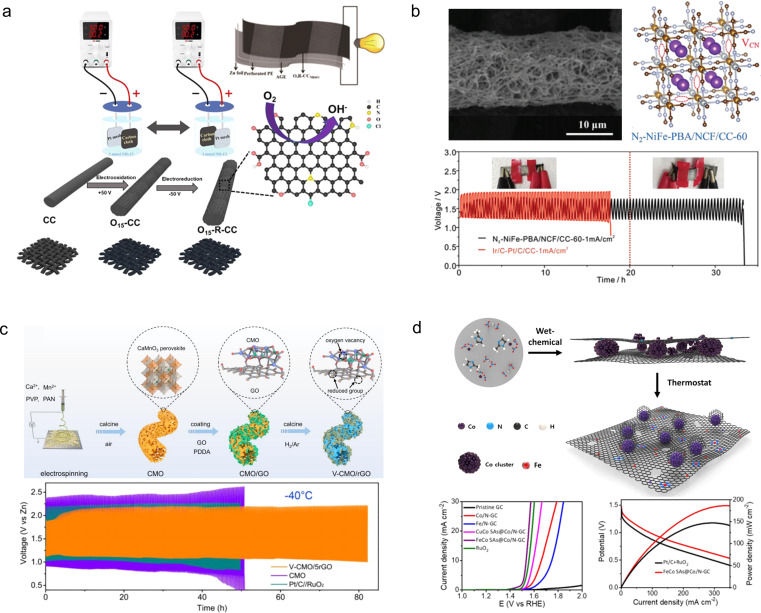
(a) Schematic diagram illustration of the electro-activation of carbon cloth.^133^ (b) Schematic diagram of the synthesis of N_2_–NiFe-PBA/NCF/CC-60 and cycle stability.^134^ (c) Schematic diagram of the synthesis process of the V-CMO/rGO.^135^ Reproduced with permission from ref. 135. Copyright 2023, Wiley-VCH GmbH. (d) Fabrication scheme of FeCo SAs@Co/N-GC catalysts.^136^ Reproduced with permission from ref. 136. Copyright 2021, American Chemical Society.

Carbon cloth, composed of a cluster of carbon filaments, exhibits remarkable electrical conductivity, mechanical strength, and flexibility, causing it to be extensively employed in flexible ZABs. Nevertheless, commercially available pristine carbon cloths typically exhibit limited specific surface areas and lack electrochemically active sites on their surfaces.^132^ In order to enhance the electrochemical properties of carbon cloth for direct utilization as a catalyst-free air cathode material in flexible ZABs, Zhang *et al.*^133^ developed a highly electrochemically active carbon cloth (Fig. 14a). The activated carbon cloth was prepared through simple electrochemical oxidation/reduction of commercial carbon cloth in an NH_4_Cl solution. Following the electrochemical treatment, a graphene-like exfoliated carbon layer formed on the surface of the carbon cloth, introducing oxygen-containing groups and doping N and Cl atoms to significantly improve its electrochemical performance. The flexible ZABs assembled on the treated carbon cloth exhibited an energy density of up to 690 W h kg^−1^ with no degradation under the bending state.

To further enhance the electrocatalytic performance of carbon cloths, it is imperative to introduce catalysts with exceptional electroactivity on their surface; however, this inevitably results in the incorporation of adhesives. The presence of a poorly conductive binder would cover the catalyst's surface, diminishing its reactive sites and increasing electrode impedance.^137^ Moreover, a conventional binder may degrade when exposed to air for extended periods, significantly impacting the cycle life of the air electrode. Therefore, there is an urgent need to design novel binder-free and efficient OER/ORR catalysts.^138^ Jiang *et al.*^134^ synthesized a flexible and self-supporting bifunctional cathode material by combining a Ni–Fe Prussian blue analogue with N-doped carbon fibers grown on carbon cloth (N_2_–NiFe-PBA/NCF/CC-60) (Fig. 14b). The *in situ* formation of CN vacancies in NiFe-PBA enhanced the catalytic activity of the oxygen evolution reaction (OER) while suppressing the loss of elemental Fe during the OER process. The 3D interconnecting network structure formed by NiFe-PBA and carbon fibers significantly increased specific surface area and electrical conductivity, ensuring sufficient and stable active sites for ORR/OER reactions. As a result, N_2_–NiFe-PBA/NCF/CC-60 exhibited excellent OER performance (overpotential of 270 mV at 50 mA cm^−2^) and ORR performance (0.89 V at 5 mA cm^−2^), enabling stable operation of ZABs assembled based on this electrode for up to 2000 hours. Peng *et al.*^135^ fabricated reduced graphene oxide-coated oxygen-rich void porous perovskite (CaMnO_3_) nanofibers (V-CMO/rGO) as an air electrode catalyst on a carbon cloth support (Fig. 14c). The incorporation of the graphene oxide coating enhanced the conductivity of the perovskite, thereby promoting reaction kinetics within the 3D network structure. Consequently, this catalyst achieved a half-wave potential of 0.86 V *vs.* RHE, enabling a high peak power density of 56 mW cm^−2^ and long cycling stability exceeding 80 h. Lee *et al.*^136^ reported a graphitic carbon nitride framework catalyst (FeCo SAs@Co/N-GC) for growing Fe, Co single atoms (Fig. 14d). The Co active sites were integrated by an electronic structure modulation method to enhance the OER and ORR performance of the catalyst. Compared with commercial Pt/C, FeCo SAs@Co/N-GC demonstrated a lower overpotential (0.29 V at *E*_*J*=10_) and Tafel slope (56.6 mV dec^−1^). The catalyst coated on a carbon cloth carrier can be directly used as an air cathode for flexible ZABs. The assembled solid-state flexible ZABs demonstrated an energy density of 1017 W h kg_Zn_^−1^ and an endurance of 680 cycles at 50 mA cm^−2^.

#### 5.2.2 Flexible electrolytes

The electrolyte of a typical flexible ZAB is typically composed of a polymer hydrogel, which functions as an ion transport pathway and an insulator to mitigate internal short circuits.^139^ The utilization of polymer electrolytes circumvents the issue of corrosive liquid leakage, thereby meeting the requirements for flexibility and safety in wearable electronics.^140^ However, common hydrogel electrolytes encounter several noteworthy challenges. Firstly, the distinctive semi-open system of ZABs accelerates water loss within the hydrogel, rendering it susceptible to reacting with atmospheric CO_2_.^141^ Secondly, due to moisture evaporation at high temperatures and sluggish ion transport at low temperatures, maintaining proper functionality under extreme temperature conditions becomes arduous.^142^ Lastly, devices incorporating hydrogel electrolytes suffer from significant charge/discharge polarization and exhibit limited cycle life.^143^

**Fig. 15:**
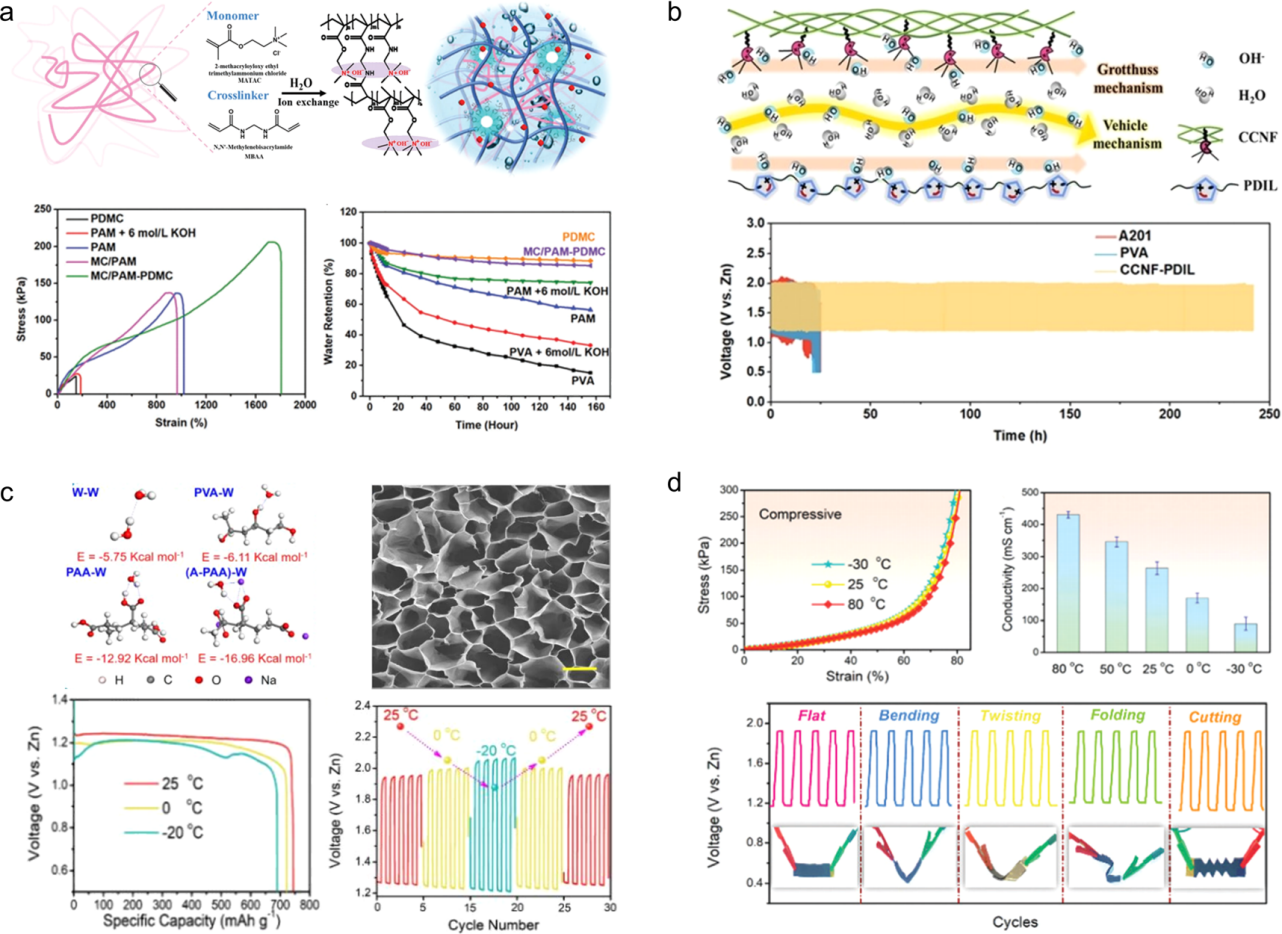
(a) PDMC ionomer network, mechanical properties, and water retention properties.^144^ Reproduced with permission from ref. 144. Copyright 2022, Wiley-VCH GmbH. (b) Schematic illustration of the ion transport mechanism *via* combination of the Grotthuss mechanism and the vehicle mechanism by synergy of channels along the CCNF surface and within PDIL.^145^ Reproduced with permission from ref. 145. Copyright 2022, Wiley-VCH GmbH. (c) Morphology and performances at low temperatures of A-PAA hydrogel.^146^ Reproduced with permission from ref. 146. Copyright 2020, Wiley-VCH GmbH. (d) Mechanical and electrochemical properties of the A-PAA hydrogel at high temperature.^147^

Taking inspiration from the intricate structure of animal dermis and the high water retention capacity of plant cells, Chen *et al.*^144^ developed a gel electrolyte with a dynamic double-permeable network structure by creating an OH^−^ conductive ionomer network *in situ* within a hollow polymer microcapsule-modified hydrogel polymer network (Fig. 15a). The resulting electrolyte exhibited excellent water retention (107 g g^−1^), ionic conductivity (215 mS cm^−1^), and ultra-high tensile strength (1800%). When used to assemble a flexible ZAB, this electrolyte demonstrated long-term cycling stability for up to 320 h without significant performance degradation even when bent at 180°.

An ionic liquid is a kind of non-volatile, high conductivity and heat stable electrolyte. It is promising to use it to design new gel electrolytes for flexible ZABs. Inspired by the reinforced concrete structure, Chen *et al.*^145^ constructed a CCNF-PDIL gel electrolyte with hierarchical nanostructures by confining the polymerization (PDIL) of a double cationic ionic liquid to a robust 3D porous cationic cellulose nanofiber (CCNF) matrix (Fig. 15b). Owing to the synergistic effect of the robust CCNF skeleton and strong noncovalent interactions, as well as the high hydrophilicity of PDIL, the CCNF-PDIL gel electrolyte exhibits high OH^−^ conductivity (286.5 mS cm^−1^), good mechanical properties (withstanding 105 MPa tensile stress), and appropriate water retention (27 g g^−1^). Assembled from CCNF-PDIL gel electrolyte ZABs have realized the cycle life of 240 h.

Although the newly designed and prepared hydrogel electrolyte exhibits excellent mechanical properties and electrical conductivity, it is susceptible to deactivation under extreme temperature conditions.^148^ Chen *et al.*^146^ developed a cold-resistant hydrogel electrolyte, demonstrating that the polarity of the end group plays a crucial role in enhancing its antifreeze performance (Fig. 15c). Motivated by this finding, they significantly increased the interaction energy between alkalized polyacrylic acid (A-PAA) and water molecules by neutralizing the carboxyl group into salt using an alkali. Consequently, the assembled flexible air cell maintained 92.7% of its initial capacity at −20 °C while retaining satisfactory flexibility. In contrast, Chen *et al.*^147^ discovered that the incorporation of a conventional low-vapor-pressure glycerol substance could ameliorate the desiccation of the water-saturated A-PAA hydrogel, thereby augmenting the stability of the hydrogel under elevated temperatures (Fig. 15d). Consequently, flexible zinc–air batteries fabricated with the modified A-PAA hydrogel can function seamlessly at temperatures as high as 80 °C.

### 5.3 Al–air batteries

Aluminium is the most abundant metal on earth, with a low redox potential (−1.67 V *vs.* SHE), high volumetric capacity (8056 mA h cm^−3^), low cost, and safety in aqueous solutions.^149^ Therefore, AABs have received increasing attention. However, the low activity of the air electrode and the formation of by-products, Al oxide/hydroxide or hydrogen, lead to a low practical specific energy of ∼1000 W h kg^−1^.^150^ Therefore, the development of efficient air cathode catalysts is of great importance.

**Fig. 16:**
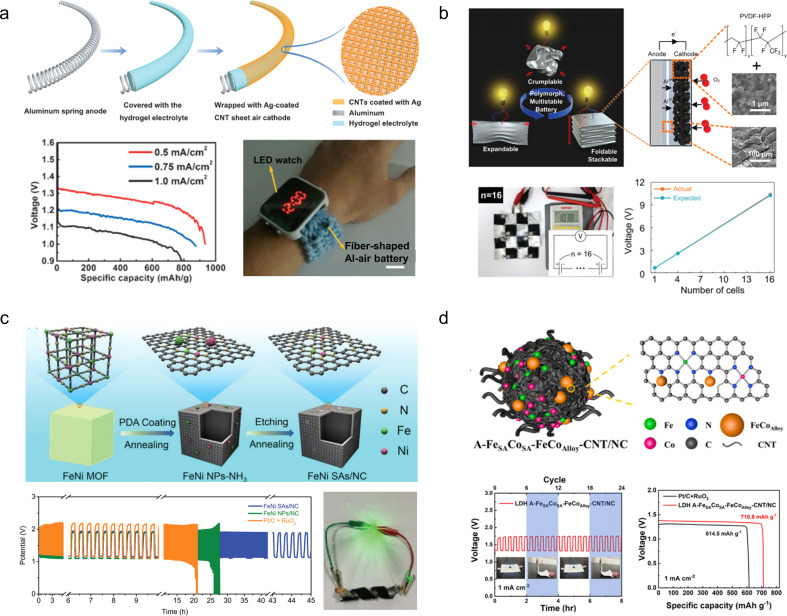
(a) Schematic diagram of the preparation and performance of the fibrous Al–air batteries.^151^ Reproduced with permission from ref. 151. Copyright 2016, Wiley-VCH GmbH. (b) Concept drawing of a shape-reconfigurable Al–air battery and the output voltage of 16 cell units in series.^152^ Reproduced with permission from ref. 152. Copyright 2017, Wiley-VCH GmbH. (c) Schematic of FeNi SAs/NC catalyst preparation, cycle stability and electronic photographs of the flexible device.^153^ Reproduced with permission from ref. 153. Copyright 2021, Wiley-VCH GmbH. (d) Schematic diagram of the NiFe LDH A–Fe_SA_Co_SA_–FeCo_alloy_–CNT/NC cathode catalyst structure and electrochemical properties.^154^ Reproduced with permission from ref. 154. Copyright 2023, Elsevier.

Peng *et al.*^151^ fabricated a fibrous AAB by sequentially coating a gel electrolyte and wrapping cross-stacked carbon nanotube/silver-nanoparticle hybrid sheets as the air cathode onto a spring-like Al substrate, achieving a specific capacity of 935 mA h g^−1^ and an energy density of 1168 W h kg^−1^. The fiber shaped AAB exhibits flexibility and stretchability, rendering it suitable for integration into wearable textiles and large-scale applications (Fig. 16a). To enhance the deformation performance of AABs, Shim *et al.*^152^ proposed a shape-reconfigurable cell for assembling flexible AABs with deformable components made of micro-meter scale or nanoscale composite materials. They fabricated an Al foil anode and a carbon composite cathode using cellulose as a flexible current collector, enabling the assembled device to withstand folding, wrinkling, and 3D expansion. The AAB demonstrates a specific capacity of 496 mA h g^−1^ and can deliver a voltage of 10.3 V when connected in series with 16 cells (Fig. 16b).

The improvement of the flexible AAB cathode catalyst performance has also been documented. For instance, Peng *et al.*^153^ synthesized a N-doped carbon matrix embedded with bimetallic MOF-derived Fe–Ni nanoparticles (FeNi SAs/NC). Density functional theory calculations revealed the presence of a stable Fe–Ni–N_6_ structure in this system, which exhibited enhanced stability and high intrinsic electrocatalytic activity. Furthermore, the hollow porous carbon matrix facilitated improved contact between the catalyst and electrolyte, leading to enhanced atomic utilization. As a result, FeNi SAs/NC demonstrated remarkable ORR activity (with an onset potential of 0.98 V and half-slope potential of 0.84 V). When incorporated into an AAB configuration, it displayed a specific capacity of 1576.4 mA h g^−1^ and maintained normal operation even under bending conditions (Fig. 16c). Li *et al.*^154^ synthesized the air cathode catalyst material (NiFe LDH A–Fe_SA_Co_SA_–FeCo_alloy_–CNT/NC) by immobilizing an iron–cobalt alloy and iron–cobalt double single-atoms in a N-doped carbon framework that contains carbon nanotubes, which exhibited a half-wave potential of 0.84 V and an OER potential of 1.56 V (*E*_*J*_ = 10 mA cm^−2^). The excellent electrocatalytic performance of this catalyst can be attributed to the augmentation of charge density on the material surface by metal/alloy nanoparticles, as well as the synergistic effect between Fe–Co dual single atoms and Ni–Fe LDH. An AAB assembled using this catalyst achieved a power density of 145 mW cm^−2^ at a current density of 157 mA cm^−2^ (Fig. 16d).

In summary, the primary challenges faced by flexible metal–air batteries are how to mitigate metal anode fracture during device deformation and maintain electrolyte stability in air. Utilizing gel electrolytes with excellent mechanical stability is a favourable option for safeguarding the metal anode against mechanical fractures and reducing its corrosion rate. Additionally, further advancements are required in developing all-solid-state metal–air batteries to prevent degradation of electrochemical properties caused by exposure to air.

The electrolytes utilized in the flexible aqueous energy storage devices (SCs, ZIBs, and metal–air batteries) are hydrogel electrolytes that possess non-volatile and non-flammable properties. Consequently, there is no risk of fire or explosion resulting from electrolyte leakage or device short-circuiting. Moreover, even if these energy storage devices endure mechanical damage caused by external forces, the internal materials can be easily collected and recycled without any significant impact on human health or the environment. Additionally, it is crucial to consider the biocompatibility of flexible electronic devices intended for prolonged interaction with human skin as this greatly influences user experience. Therefore, it is imperative to fabricate an encapsulation shell for such devices that exhibits skin-friendly characteristics. However, there remains a scarcity of relevant research in this field at present.

## 6. Flexible lithium-ion batteries

Since its introduction in 1991, LIBs with high energy density and stable output voltage have become the primary power supply system for everyday electronic products.^155^ The energy storage mechanism of LIBs is similar to that of ZIBs. By facilitating the transmission of Li^+^ ions between electrolytes and enabling the intercalation/deintercalation process of the cathode and anode, the charging and discharging process of LIBs can be achieved.^156^ When used in wearable electronic devices, LIBs ensure prolonged durability.^157,158^ However, due to the utilization of organic electrolytes in these batteries, it is essential to consider safety aspects during the development of flexible electrodes and electrolytes while ensuring stability amidst device deformation.^159,160^

### 6.1 Flexible electrodes

#### 6.1.1 Foldable electrodes

**Fig. 17:**
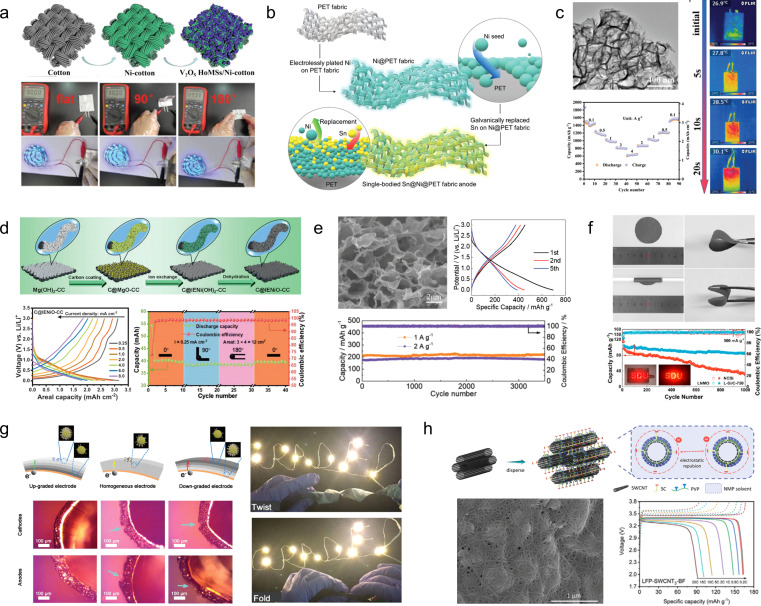
(a) Schematic of the fabrication process of the V_2_O_5_ hollow multishelled structures/Ni-cotton fabric electrode and the electrode performances. Reproduced with permission.^161^ Reproduced with permission from ref. 161. Copyright 2020, Wiley-VCH GmbH. (b) A schematic of the stepwise fabrication of the single-bodied Sn@Ni fabric electrode. Reproduced with permission.^162^ Reproduced with permission from ref. 162. Copyright 2020, Wiley-VCH GmbH. (c) CoP@CC anode.^163^ Reproduced with permission from ref. 163. Copyright 2022, Wiley-VCH GmbH. (d) Schematic illustration of fabrication of the C@IENiO-CC freestanding electrode.^164^ Reproduced with permission from ref. 164. Copyright 2021, Wiley-VCH GmbH. (e) TEM and performances of 3D porous MXene foams.^165^ Reproduced with permission from ref. 165. Copyright 2021, Wiley-VCH GmbH. (f) Digital photographs and cycle stability of MXene/L-Si/C.^166^ Reproduced with permission from ref. 166. Copyright 2020, American Chemical Society. (g) The gradient distribution of binders enables simultaneous optimization of bendability and electronic conductivity in the upgraded electrode.^167^ Reproduced with permission from ref. 167. Copyright 2022, Wiley-VCH GmbH. (h) Schematic diagram, SEM and rate performance of LFP/SWCNTs.^168^

Due to excellent chemical stability, electrical conductivity and bendability, textile-based energy storage devices have garnered attention for applications in flexible and wearable electronics. Wang *et al.*^161^ employed the solution-treated polymer-assisted metal deposition method to prepare a Ni-cotton flexible substrate, onto which they coated V_2_O_5_ with a hollow multi-shell structure to fabricate the V_2_O_5_ hollow multishelled structures/Ni-cotton composite electrode (Fig. 17a). Taking advantage of the desirable structure of V_2_O_5_ HoMSs that effectively buffers the volume expansion and alleviates the stress/strain during repeated Li-insertion/extraction processes. Consequently, the capacity remains at 222.4 mA h g^−1^ after 500 charge–discharge cycles, and it can withstand hundreds of bending cycles. However, the Ni-cotton flexible substrate serves solely as a current collector. To further enhance the electrochemical performance and deformability of the electrode material, Lee and his colleagues^162^ took advantage of the electrochemical potential difference between Ni and Sn to replace the thin layer of metal Ni deposited on the flexible fabric with Sn to obtain a single-bodied Sn@Ni fabric electrode (Fig. 17b). The electrode structure possesses a bi-continuous ion/electron transport path, and the Sn volume changes driven by the Ni matrix are also alleviated during repeated lithiation/de-lithiation. Consequently, both the electrochemical and mechanical properties of the electrode are synergistically improved.

In addition to metallic fabrics, low-cost and easy-to-handle 2D carbon materials can also serve as suitable flexible substrates. When used as a substrate for transition metal compound anode materials with high theoretical specific capacity, it can significantly expedite lithium-ion diffusion and enhance electron transport kinetics, thereby optimizing the multiplicity performance and cycling stability of the active materials. Yang *et al.*^163^ successfully synthesized arrays of CoP nanosheets on carbon cloth through a facile one-step electrodeposition and *in situ* phosphorylation strategy, which served as a binder-free anode material for LIBs (Fig. 17c). The CoP nanosheets exhibited excellent interfacial bonding properties with the carbon cloth substrate, effectively preventing agglomeration and exposing more reaction sites. Moreover, the 3D lattice morphology of the electrodeposited CoP nanosheets provided sufficient space to accommodate volume expansion during charging. The CoP@CC demonstrated a high discharge capacity of 1866.9 mA h g^−1^ (3.8 mA h cm^−2^). Furthermore, the assembled full-battery device exhibited slow warming rates during external short circuits, ensuring enhanced safety. In comparison to the conventional direct carbon coating method, Lu *et al.*^164^ employed an indirect carbon cladding approach to synthesize a carbon-covered NiO composite material on a carbon cloth (Fig. 17d). This novel method ensured the formation of a well-graphitized carbon cladding layer while preventing the reduction of transition metal oxides into metal monomers at elevated temperatures. The resulting anode composites exhibited a high areal capacity of 3.08 mA h cm^−2^ at 0.25 mA cm^−2^ and demonstrated excellent flexibility.

The 2D transition metal carbides and nitrides, called MXene, are currently under development as anode materials for flexible LIBs. Xu *et al.*^165^ successfully fabricated 3D MXene foams with a well-defined porous structure using a simple sulfur templating method, which effectively prevents the aggregation and re-stacking of MXene nanosheets while providing abundant active sites for Li^+^ storage (Fig. 17e). The resulting self-supporting foam exhibits excellent bendability and high electrical conductivity, delivering a capacity of 455.5 mA h g^−1^ at 50 mA g^−1^ when directly employed as a LIB anode. Moreover, it demonstrates outstanding rate performance and long-term cycling stability. Feng *et al.*^166^ synthesized the layered Si/C materials using MXene films as flexible substrates (Fig. 17f). Incorporating carbon with a reasonable degree of graphitization can effectively mitigate the volume expansion of Si and enhance its conductivity. Moreover, the integration of monolayer Si and C facilitates the transport of Li^+^ within the active material. Consequently, this composite material exhibits remarkable mechanical robustness while maintaining a capacity retention of 82.85% over 3200 cycles at 5 A g^−1^.

Layered oxides (LiNi_1−*x*_M_*x*_O_2_, M = Co, Mn, and Al), spinel oxides (LiNi_0.5_Mn_1.5_O_4_), and polyanionic compounds (phosphates, sulfates, silicates) play a crucial role as cathode materials in LIBs.^169^ In the process of assembling flexible LIBs, they are generally coated on flexible conductive substrates as active materials. In general, the easiest way to increase the energy density of a battery is to increase the load of the active material. Nevertheless, such electrodes typically become more susceptible to mechanical fracture under deformation conditions like bending and folding compared to thin and lightweight electrodes with lower loading.^170^ Consequently, designing flexible LIBs that strike a balance between mechanical properties and energy density becomes challenging. To tackle this issue, Chen and his colleagues^167^ proposed a mechanically graded electrode featuring a gradient distribution of maximum allowable strain to mitigate the mismatch between the mechanical properties of the electrode material and energy density (Fig. 17g). The LiNi_1/3_Mn_1/3_Co_1/3_O_2_ cathode fabricated based on this concept demonstrated exceptional flexibility withstanding a bending radius of 400 μm, while maintaining 92.3% of its initial performance after undergoing 550 consecutive bending tests. Wang *et al.*^168^ developed a method for the massive production of ultra-long single-walled carbon nanotubes in *N*-methyl-2-pyrrolidone solution by harnessing electrostatic dipole interactions and spatial site resistance of dispersant molecules, while incorporating a low concentration of LiFePO_4_ as a conductive additive (Fig. 17h). The composite electrode material exhibits a stress tolerance of 7.2 MPa and can withstand various degrees of bending and folding. A high-quality loaded electrode with a mass loading of 39.1 mg cm^−2^ was successfully fabricated, demonstrating rate capacities of 161.5 mA h g^−1^ at 0.5C and 130.2 mA h g^−1^ at 5C, respectively, while maintaining a capacity retention rate of 87.4% after undergoing 200 cycles at 2C.

#### 6.1.2 Stretchable electrodes

**Fig. 18:**
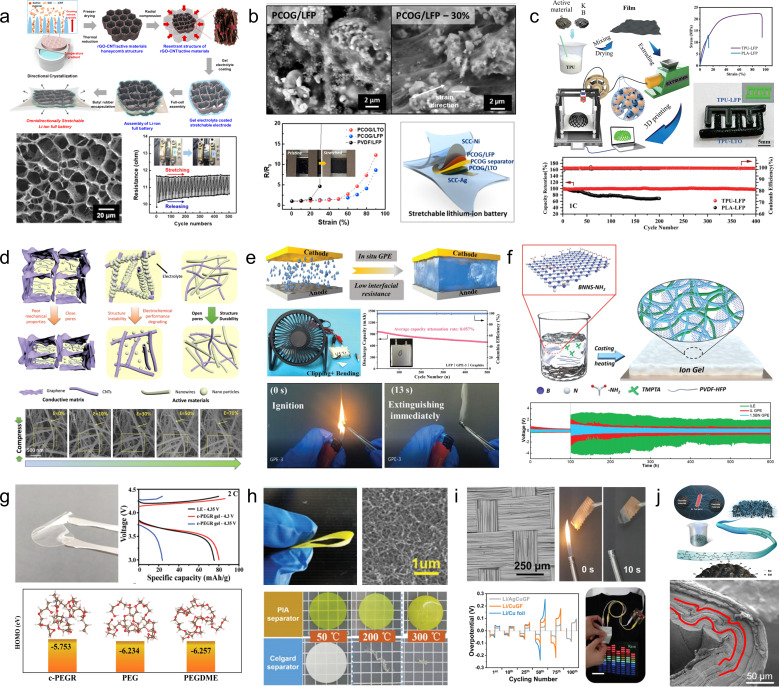
(a) Schematic illustration of the fabrication of the stretchable lithium-ion battery based on micro-honeycomb structures of graphene–carbon nanotube (CNT)/active materials.^171^ Reproduced with permission from ref. 171. Copyright 2020, American Chemical Society. (b) Scanning electron microscope images and mechanical properties of pristine PVDF/LFP.^172^ Reproduced with permission from ref. 172. Copyright 2022, American Chemical Society. (c) Schematic illustration of the fabrication process of the 3D-printed TPU-based electrodes.^173^ Reproduced with permission from ref. 173. Copyright 2023, Wiley-VCH GmbH. (d) Schematic illustration of different types of 3D compressible electrodes.^174^ Reproduced with permission from ref. 174. Copyright 2021, Wiley-VCH GmbH. (e) Preparation and characterization of GPE.^175^ Reproduced with permission from ref. 175. Copyright 2021, Wiley-VCH GmbH. (f) Schematic diagram of the preparation process of functionalized BN nanosheet added ion gel.^176^ Reproduced with permission from ref. 176. Copyright 2020, Wiley-VCH GmbH. (g) Optical photograph and characterization of a c-PEGR gel.^177^ (h) High flexibility and fire resistance of the PIA separator.^178^ Reproduced with permission from ref. 178. Copyright 2021, Wiley-VCH GmbH. (i) The characterization of MGFs.^179^ Reproduced with permission from ref. 179. Copyright 2023, Wiley-VCH GmbH. (j) Schematics and morphology characterization of the SBCIF.^180^ Reproduced with permission from ref. 180. Copyright 2022, American Chemical Society.

Stretchable electrode materials are the core components of stretchable LIBs, which determines the electrochemical properties of stretchable devices. However, the active materials for LIBs tend to be rigid materials and require corresponding deformable binders and inactive stretchable substrates to achieve the overall electrode stretchability. Therefore, preparing stretchable electrode materials is a relatively challenging task. For example, Son *et al.*^171^ designed a composite electrode consisting of concave-angle honeycomb graphene–carbon nanotubes/LiPF_6_ (Fig. 18a). The entangled CNTs and graphene sheets formed an interconnected network framework, providing mechanical stability and allowing structural stretching during deformation within the inwardly protruding framework of the concave honeycomb structure. This design effectively avoided energy density loss caused by using an inactive elastic substrate. Additionally, the gel electrolyte located inside the honeycomb structure enhanced mechanical stability in the stretched state while serving as a stretchable spacer. The electrode demonstrated an area capacity of 5.05 mA h cm^−1^ and maintained a capacity retention of 95.7% after undergoing 100 cycles of stretching. Son *et al.*^172^ have developed a fully integrated, stretchable, and printable LIB system that encompasses electrodes, collectors, and diaphragms (Fig. 18b). The electrode LiFePO_4_ was modified with a physically crosslinked organo-gel derived from controlled crystal growth of vinylidene fluoride bias to achieve improved adhesion to the active material while maintaining stretchability. Even after 5000 cycles at 50% strain, the composite electrode material exhibited only a marginal increase in impedance, indicating exceptional electrochemical stability during stretching. The fabric-based assembled stretchable LIBs demonstrated a capacity of 132 mA h g^−1^ under conventional conditions (0.5C), with an impressive capacity retention of 88% at 50% stretching. Moreover, these batteries can be seamlessly integrated into wearable electronic devices.

The 3D printing technology demonstrates significant potential in the production of flexible and customizable high-performance LIBs, which are essential for the upcoming era of smart and ubiquitous energy. However, there still exists a notable performance disparity between 3D printed electrodes and conventional electrodes, particularly regarding cycling stability. This limitation severely hinders the practical implementation of 3D printed batteries. To address this issue, Chen *et al.*^173^ have developed a series of intricate 3D printed electrodes based on thermoplastic polyurethane (TPU) (Fig. 18c). By leveraging TPU's exceptional stress-buffering properties, these electrodes not only exhibit adaptability to volume changes during charge–discharge cycling and prevent microstructure collapse in 3D printed electrodes but also demonstrate remarkable resistance to significant bending angles. Moreover, they showcase an impressive capacity retention rate close to 100% after undergoing 300 cycles at a rate of 1C, thereby providing novel insights for bridging the performance gap between 3D printed batteries and conventional LIBs.

#### 6.1.3 Compressible electrodes

Cao *et al.*^174^ have successfully synthesized open-cell dual-mesh sponges comprising two interconnected networks of highly conductive carbon nanotubes and TiO_2_ nanowires embedded with Li^+^ through chemical vapor deposition followed by a hydrothermal method, aiming to simultaneously achieve enhanced energy density and mechanical stability (Fig. 18d). The 1D components within the sponge effectively interconnect with each other, resulting in a cross-linked network that imparts excellent compressibility to the electrode. The specific capacity (240 mA h g^−1^) remains virtually unchanged even after subjecting the electrode to 1000 cycles of compression at a strain of 50%. However, conventional carbon foam-based compressible electrodes often fail to withstand folding and tensile deformation, thereby imposing significant limitations on their practical utilization in battery devices, restricting their application solely to pressure sensors.

### 6.2 Flexible electrolytes, separators, and current collectors

The utilization of flexible LIBs in wearable electronic devices inevitably entails the risk of electrolyte leakage, which is further compounded by the flammable and explosive properties of commonly employed organic electrolytes. To enhance the safety of flexible LIB electrolytes, an innovative design approach becomes necessary. The primary research concept revolves around designing polymer gel electrolytes as alternatives to conventional liquid counterparts; however, this often leads to a compromise in the electrochemical performance of LIBs, thereby presenting ongoing challenges in the design and preparation of electrolytes for flexible applications.

Hu *et al.*^175^ developed a non-flammable gel polymer electrolyte that effectively addresses the fundamental issue of battery safety (Fig. 18e). The gel electrolyte (GPE-3) was synthesized through *in situ* polymerization of tri(acryloyloxyethyl) phosphate and triethylene glycol dimethacrylate within the electrolyte solution, with only 7.5 wt% flame retardant added to ensure minimal impact on electrochemical performance. Even when exposed to fire for over 10 s, GPE-3 remained non-combustible. Furthermore, graphite//GPE-3//LiFePO_4_ cells exhibited normal operation under various deformation conditions and demonstrated exceptional long-term cycling stability (average capacity degradation rate: 0.057%).

In addition to the necessity of ensuring electrolyte safety, the electrolyte also plays a pivotal role in ion diffusion rate within batteries. Despite their exceptional mechanical properties, polymer gels often suffer from sluggish diffusion kinetics, which consequently impacts overall device capacity. To address this concern, Chen and colleagues^176^ developed ionic gel electrolytes incorporating amine-functionalized boron nitride nanosheets (AFBNNSs) through thermal polymerization processes, achieving an impressive maximum tensile strain of 72% with a tensile strength of 2.22 MPa (Fig. 18f). Importantly, the strong interaction between amine groups on boron nitride nanosheets and bis(trifluoromethanesulfonyl)imide anions significantly reduces anion mobility while enhancing Li^+^ mobility. LIBs assembled using this electrolyte demonstrated stable lithium deposition for over 600 hours. Wang *et al.*^177^ have synthesized cross-linked poly(ethylene glycol)-based resin (c-PEGR) gel electrolytes prepared by the ring-opening reactions of poly(ethylene glycol)diglycidyl ether (PEGDE) with epoxy groups and poly(ether amine) (PEA) with amines (Fig. 18g). These gel electrolytes exhibited exceptional Li^+^ transport and compatibility, along with enhanced flexibility and improved oxidative stability. Due to the confinement of hydroxyl groups within the crosslinked structure, their movement is restricted, resulting in an oxidation potential of 4.36 V. Consequently, this c-PEGR gel electrolyte holds great promise for utilization in high-voltage flexible LIB assemblies.

In addition to the electrolyte, the separator and current collector are pivotal components of LIBs, exerting a critical influence on both thermal safety and electrochemical stability; however, there is a paucity of research in this domain. For example, Cheng *et al.*^178^ fabricated a novel polyimide aerogel (PIA) separator with high-temperature resistance through spin-coating, which exhibits excellent foldability and bendability without any damage (Fig. 18h). The PIA diaphragm possesses the remarkable characteristics of an aerogel, including a high porosity of up to 78.35%, thereby enhancing its wettability. Moreover, its unique 3D mesh structure can significantly improve the rate capability and cycling stability of LIBs. Additionally, the chemical cross-linking method employed in the preparation process has successfully enhanced its thermal stability (>500 °C). In summary, this gel-based diaphragm holds great potential for flexible LIB applications.

Current collectors are crucial components that facilitate electron transport and provide mechanical support for the electrodes. However, since they do not contribute to the capacity, minimizing the mass of the current collector enhances the mass energy density of the cell. In line with this concept, Zheng *et al.*^179^ introduced a novel type of low-density (2.9 mg cm^−2^) current collector composed of 3D metal glass-fiber fabrics (MGFs) (Fig. 18i). These MGFs were fabricated using various metal foils (Cu, Ni, Ag), exhibiting exceptional tensile strength (>150 MPa) and negligible impedance alteration even after enduring 100 000 bending cycles. Moreover, their flexibility was significantly improved compared to conventional metal foils due to their pliable nature. Additionally, owing to the fire-resistant properties conferred by glass fibers, MGFs exhibit non-combustibility even at surface temperatures as high as 500 °C. Furthermore, these collectors demonstrate electrochemical compatibility with a wide range of mainstream LIB cathodes and anodes. Deng *et al.*^180^ proposed a simple and versatile bio-self-growth strategy for the preparation of flexible LIBs (Fig. 18j), in which the electrodes and the diaphragm (SBCIF) are essentially integrated into a single whole, capable of adapting to various active materials. Meanwhile, owing to the good self-generated corrugated structure that helps to disperse the stress, no rupture or electrode/diaphragm separation was observed after 20 000 bending cycles at a bending angle of 165° and a bending radius of 3 mm. The flexible LIBs assembled based on SBCIF maintained 95% of their initial capacity after 10 000 bending/compression cycles.

In summary, to design deformable LIBs with both mechanical and electrochemical properties, it is essential to employ electrically active flexible substrates for electrode material preparation or directly design the active materials to be deformable. Furthermore, enhancing the synergistic effect of the composite is crucial. Additionally, while ensuring the device's electrochemical performance remains uncompromised, efforts should be made to maximize fire resistance in electrolyte design and eliminate safety hazards.

## 7. Flexible lithium–sulfur batteries

The unstable electrochemical properties and high flammability of LIBs under mechanical deformation conditions have hindered the widespread utilization of LIBs in flexible electronics.^181^ LSBs are anticipated to replace LIBs due to their high economic viability, non-toxic nature, and abundant availability of active materials for cathodes.^47,182^ Considering the two-electron system during the complete electrochemical reaction, the sulfur cathode exhibits a theoretical capacity of up to 1672 mA h g^−1^, which is approximately 2 times higher than that of conventional LIB active materials.^183^

However, challenges persist in the application of LSBs for flexible energy storage. Firstly, the volumetric changes experienced by the sulfur cathode during charge and discharge processes result in continuous capacity degradation.^184^ Secondly, the low conductivity exhibited by the sulfur cathode leads to sluggish electrochemical kinetics.^185^ Lastly, the dissolution of polysulfides in electrolytes gives rise to the phenomenon known as the “shuttle effect”.^186^ In order to tackle these issues, researchers have made efforts towards designing and synthesizing various novel sulfur cathodes, separators, and electrolytes.

### 7.1 Flexible electrodes

Despite the high theoretical specific capacity and energy density of LSBs, sulfur cathodes still encounter several challenges, including low conductivity, instability of conventional polymer binders, and the shuttling phenomenon induced by Li_2_S_*x*_ in the electrolyte.^187^ Therefore, when designing cathode materials for flexible LSBs, it remains imperative to address these aforementioned issues in order to optimize their electrochemical performances. The preparation of sulfur cathodes has been attempted using carbon-based and conductive polymer-based materials.

#### 7.1.1 Carbon-based cathodes

**Fig. 19:**
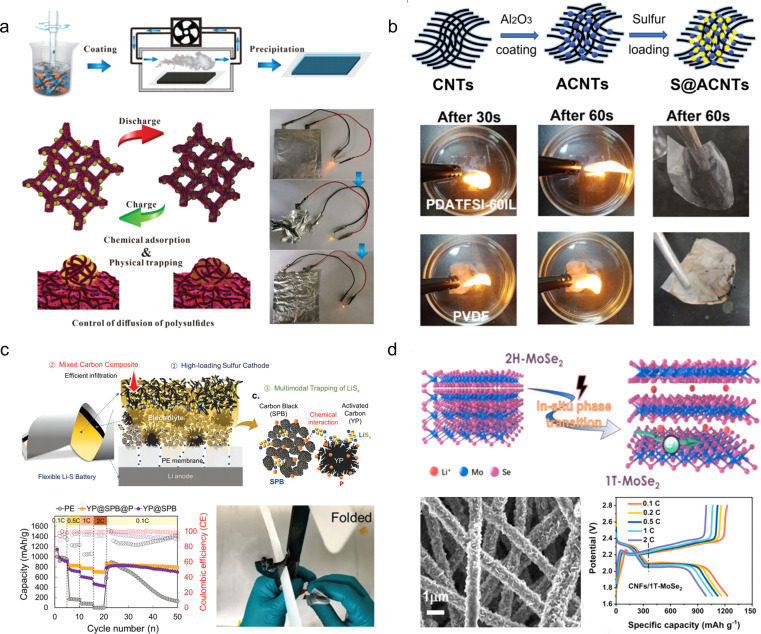
(a) Designed functions of the electrode skeleton for addressing the critical issues of volume change and LiPS shuttle effects in composite sulfur electrodes.^190^ (b) Synthesis process and high temperature resistance test of S@ACNTs materials.^191^ Reproduced with permission from ref. 191. Copyright 2022, Wiley-VCH GmbH. (c) Structures of the flexible LSBs and infiltration behaviour of the liquid electrolyte on a PE membrane.^192^ Reproduced with permission from ref. 192. Copyright 2022, Wiley-VCH GmbH. (d) Schematic diagram of MoSe_2_*in situ* phase transformation processes, and its morphology and properties.^193^

Carbon materials with high conductivity and graded porous structure are favourable for ion transport channels for polysulfide capture in LSBs. Therefore, various carbon-based materials can be employed to modify sulfur cathodes.^188,189^ For instance, Yang *et al.*^190^ introduced a self-assembled electrode paste comprising a well-designed two-component (polyacrylonitrile and polyvinylpyrrolidone) polar binder and carbon nanotubes. Both the experimental and simulation results demonstrate that the resulting 3D electrode skeleton forms an exceptionally robust and mechanically flexible electronically conducting network with a hierarchical porous structure encompassing macropores and micropores, thereby providing ion transport channels, enhanced electrolyte wetting, and efficient polysulfide trapping capabilities. Following 1000 charge–discharge cycles at 0.5C, the specific capacity stabilized at 485 mA h g^−1^ (Fig. 19a). Kim *et al.*^191^ synthesized sulfur-loaded Al_2_O_3_ carbon nanotubes (S@ACNTs) and fabricated the cathode using a solid-state electrolyte with a PDATFSI binder (Fig. 19b). The resulting full cell demonstrated remarkable characteristics including high Li^+^ conductivity of 0.45 mS cm^−1^ at room temperature, excellent thermal stability up to 450 °C, strong bond strength with an Al collector up to 24 MPa, sustainable nonflammability, and desirable flexibility. Moreover, the electrode exhibited outstanding performance in terms of capacity retention (only 12.3% decay from 0.2C to 2C) and cycling stability (91.7% capacity retention after 200 cycles). Park *et al.*^192^ fabricated freestanding carbon nanotube cathodes loaded with high-sulfur content, incorporating phosphorus-doped carbon interlayers on commercial diaphragms. The integration of an active material and structural design strategies in the electrodes and diaphragms effectively immobilized Li polysulfides through a multi-peak trapping effect, resulting in significantly enhanced electrochemical performance in terms of redox kinetics and cycling stability. The foldable LSBs demonstrated a stable specific capacity of 850 mA h g^−1^ after 100 cycles, exhibiting exceptional mechanical flexibility even under severe deformation conditions without any occurrence of short-circuit or failure (Fig. 19c). Zhang *et al.*^193^ achieved a precise controllable phase transition from 2H MoSe_2_ to 1T MoSe_2_ on flexible carbon fibers. The resulting CNFs/1T-MoSe_2_ exhibits high charge density, robust polysulfide adsorption capabilities, and efficient catalytic kinetics. Furthermore, the LSBs based on this composite material demonstrates an impressive capacity retention of 875.3 mA h g^−1^ after undergoing 500 cycles at a rate of 1C, while maintaining an area capacity of 8.71 mA h cm^−2^ even under high sulfur loading conditions of 8.47 mg cm^−2^ (Fig. 19d).

#### 7.1.2 Conducting polymer-based cathodes

**Fig. 20:**
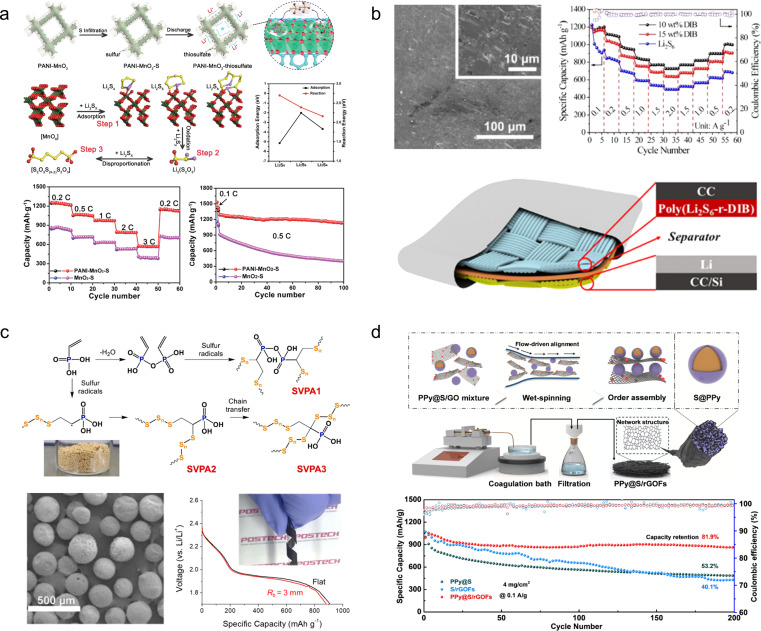
(a) Schematic illustration of polysulfides anchored by PANI-MnO_2_ to form a thiosulfate (polythiosulfate) complex and the electrochemical properties.^195^ (b) Digital images and rate performance of the Li_2_S_6_-rDIB.^196^ Reproduced with permission from ref. 196. Copyright 2021, American Chemical Society. (c) Morphology and properties of the SVPA cathode.^197^ Reproduced with permission from ref. 197. Copyright 2021, Elsevier. (d) The microfluidic assembly process and cycle stability of PPy@S/rGOFs.^198^ Reproduced with permission from ref. 198. Copyright 2022, Wiley-VCH GmbH.

Carbon-based cathode materials could significantly enhance the mechanical properties and cycling stability of LSB cathodes. However, carbon-based materials with non-polar characteristics fail to completely suppress the “shuttle effect” of LiPSs. In contrast, conducting polymers such as polypyrrole (PPy), polyaniline (PANI), and poly(3,4-ethylenedioxythiophene) (PEDOT) possess conjugated structures and polar functional groups that not only enhance their electrical conductivity but also exhibit favourable thiophilicity.^194^ Consequently, numerous conductive polymers have been successfully employed as coating materials for sulfur cathodes. For instance, Su *et al.*^195^ fabricated an ultrathin and flexible layer of polyaniline with modified MnO_2_ nanoparticles through *in situ* self-assembly (Fig. 20a). The resulting 3D hierarchical porous network not only facilitated volume expansion mitigation but also expedited the transfer of reactive species, electrons, and ions by providing rapid channels, thereby enhancing the kinetics of redox reactions. Following 100 cycles at 0.5C, the initial discharge capacity reached 1275 mA h g^−1^ while maintaining a stabilized reversible capacity of 1195 mA h g^−1^; even under a high current density of 2C, the capacity could still reach 640 mA h g^−1^ after undergoing 500 discharges. Zheng *et al.*^196^ synthesized a prelithiated polysulfide-based copolymer, poly(Li_2_S_6_-r-1,3-diisopropenylbenzene), through a straightforward copolymerization reaction between Li_2_S_6_ and 1,3-diisopropenylbenzene (Fig. 20b). This copolymer was employed as a cathode material for LSBs. The authors discovered that Li_2_S_6_ forms strong chemical bonds with the conjugated aromatic backbone of the copolymer, effectively impeding polysulfide solvation. Moreover, the prelithiated polysulfide-based copolymer can facilitate Li^+^ redox kinetics by providing additional Li^+^ ions, leading to batteries exhibiting stable cycling performance and high-rate capability along with excellent coulombic efficiency. Consequently, an impressive capacity of 934 mA h g^−1^ is achieved even at a current density of 2 A g^−1^. Due to its exceptional mechanical properties, Li_2_S_6_-rDIB can be directly cast onto commercially available carbon cloth and utilized as a flexible cathode without requiring binders or conductive agents.

In the pursuit of higher specific capacity and charge/discharge rates, Park *et al.*^197^ successfully synthesized SVPA microparticles containing various sulfur isomers *via* a rapid (30 min) one-pot reaction involving elemental sulfur and vinyl phosphonic acid (Fig. 20c). The well-ordered sulfur domains within these microparticles contribute to the formation of a large surface area, thereby enhancing the lithium diffusion coefficient and facilitating polysulfide conversion kinetics in Li–S batteries. Moreover, the abundant presence of phosphonate molecules on both the surface and interface of these microparticles serves as effective chemical anchors for lithium polysulfides, effectively mitigating the shuttle effect and extending cycle life at different rates. Consequently, when assembled into a full cell configuration, this system exhibits an impressive discharge capacity of 1529 mA h g^−1^ at 0.05C along with excellent rate performance (721 mA h g^−1^ at 7C). To further enhance the mechanical properties of the electrode materials, Bao *et al.*^198^ proposed a synergistic interfacial bonding enhancement strategy by uniformly implanting polypyrrole@sulfur (PPy@S) nanospheres into the built-in cavities of self-assembled reduced graphene oxide fibers (rGOFs) using a simple microfluidic assembly method (Fig. 20d). The PPy@S/rGOFs composites exhibited excellent adsorption capacity, rapid reaction kinetics, and remarkable mechanical flexibility due to the synergistically enhanced interfacial chemical bonding between the carbon interface and polymer interface. Moreover, these composites demonstrated outstanding cycling stability with 81.9% capacity retention over 200 cycles at 0.1 A g^−1^ and exceptional rate performance with a specific capacity of 523 mA h g^−1^ at 5 A g^−1^.

### 7.2 Flexible electrolytes, separators, and current collectors

**Fig. 21:**
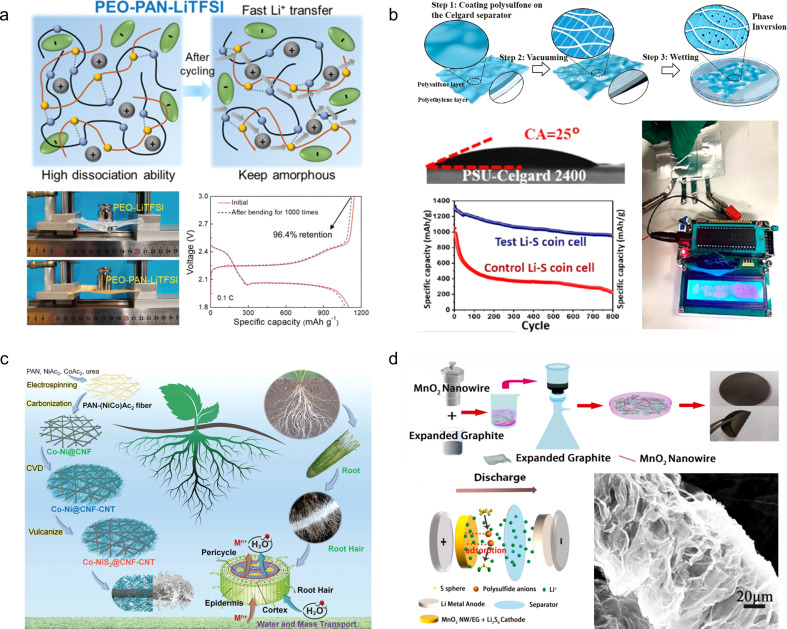
(a) Mechanical and electrochemical performance of PEO–PAN–LiTFSI.^201^ Reproduced with permission from ref. 201. Copyright 2022, Wiley-VCH GmbH. (b) Schematic of the synthetic procedures of PSU-Celgard separators.^202^ Reproduced with permission from ref. 202. Copyright 2021, American Chemical Society. (c) Schematic illustrations of the fabrication of hierarchical fibrous Co–NiS_2_@CNF–CNT with grass root hair structure as an interlayer for LSBs.^203^ Reproduced with permission from ref. 203. Copyright 2023, Wiley-VCH GmbH. (d) Schematic illustration for the synthesis of the MnO_2_ NW/EG current collector.^204^ Reproduced with permission from ref. 204. Copyright 2021, Elsevier.

Polysulfide shuttling in liquid electrolytes, lithium dendrite growth, and contact issues during bending are the primary factors contributing to capacity degradation, potential safety concerns, and limited flexibility. To address these challenges, gel polymer electrolytes are considered a superior choice for flexible LSBs due to their significantly reduced polysulfide solubility compared to liquid electrolytes, enhanced inhibition of lithium dendrite formation, and improved ability to maintain component connectivity under bending conditions.^199^ Furthermore, their lower interfacial resistance relative to ceramic solid-state electrolytes is better suited for mitigating the sluggish kinetics associated with the sulfur reaction.^200^ For example, Zhou *et al.*^201^ developed a (polyethylene oxide–polyacrylonitrile) (PEO–PAN) copolymer membrane electrolyte, where PAN fibers served as both fillers and cross-linkers (Fig. 21a). This unique structure not only enhanced the ionic conductivity, mechanical strength, and lithium dendrite suppression ability but also effectively hindered polysulfide shuttling due to the strong adsorption of polysulfides by the C=N–O functional groups formed during PEO and PAN crosslinking. Consequently, this electrolyte exhibited improved safety, cycling stability, and rate performance when employed in LSBs. The exceptional mechanical properties and bonding capability of the electrolyte enabled the flexible battery to retain over 96% of its capacity after undergoing 1000 bending cycles.

Modification of commercial diaphragms is also a viable strategy for suppressing the polysulfide shuttle effect. For instance, Chen *et al.*^202^ employed a phase transition approach to functionalize commercial diaphragms with polysulfone (Fig. 21b). The incorporation of PSU not only enhances the mechanical properties and thermal stability of the diaphragm but also improves the overall safety of the battery system. Moreover, reducing the pore size of the diaphragm effectively restricts the occurrence of the shuttle effect, thereby enabling excellent cycling stability in flexible LSBs. In fact, even in its folded state, this flexible full cell can achieve an impressive lifetime of up to 800 cycles. Inspired by the grassroots system, Chen and his colleagues^203^ designed and fabricated a flexible hierarchical carbon nanofiber–carbon nanotube membrane modified with doped NiS_2_ nanoparticles for LSB separators (Fig. 21c). DFT calculations demonstrated that Co doping induced an electron-deficient region at the doping site of NiS_2_, thereby enhancing chemisorption and catalytic activity towards lithium polysulfides. The intercalation of Co–NiS_2_@CNF–CNT in the battery exhibited exceptional performance with a high specific capacity of 951.4 mA h g^−1^ at 3C, a reversible capacity of 944.1 mA h g^−1^ after 500 cycles at 0.2C, and an impressive cycle life of up to 3000 cycles at 5C.

It is crucial to design a flexible collector that is compatible with the sulfur cathode. Shao *et al.*^204^ have developed a stable and flexible collector composed of ultra-long MnO_2_ nanowires and expanded graphite nanosheets (MnO_2_ NW/EG) with excellent electronic conductivity (Fig. 21d). This self-supporting cathode, in combination with Li_2_S_6_, demonstrates high performance for flexible LSBs. The ultra-long MnO_2_ nanowires enhance the adsorption and catalytic conversion ability of lithium polysulfides through their polar active surfaces. Moreover, the incorporation of MnO_2_ NW/EG, which exhibits remarkable mechanical flexibility, effectively mitigates the volume expansion of sulfur during charge–discharge cycles. Notably, even at a sulfur loading as high as 7 mg cm^−2^, an impressive capacity of up to 2.5 mA h cm^−2^ can be achieved.

## 8. Other flexible batteries

LIBs and LSBs are currently one of the most widely used types of batteries, but due to the shortage of Li resources and increasing costs, alternative energy storage systems such as Na^+^ ion and K^+^ ion batteries have been researched.^205^ SIBs and PIBs have shown great potential due to their relatively low cost, abundant reserves, and comparable performance to that of LIBs.^206^ However, due to the larger ionic radii of Na^+^ and K^+^ ions compared to Li ions, their diffusion in the electrolyte is impeded, resulting in diminished rate performance and cycling stability. Additionally, repeated insertion/extraction of large radius examples can cause severe volume changes in the electrode material that accelerate battery capacity decay. To solve these problems, various attempts have been made to design and prepare new flexible electrodes and electrolytes.

### 8.1 Sodium ion batteries

#### 8.1.1 Flexible electrodes

**Fig. 22:**
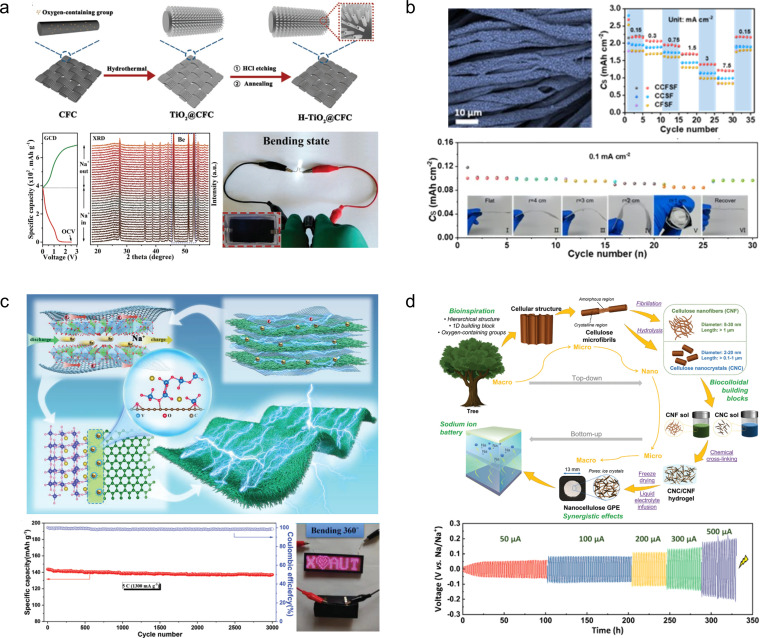
(a) Schematic illustration of the preparation of H–TiO_2_@CFC, *in situ* XRD patterns, and photograph of the battery.^207^ Reproduced with permission from ref. 207. Copyright 2020, Wiley-VCH GmbH. (b) SEM image and electrochemical properties of CCFSF.^208^ Reproduced with permission from ref. 208. Copyright 2020, Wiley-VCH GmbH. (c) Schematic diagram of the free-standing NVO–rGO heterointerface with the V–O–C bond and its anomalous Na^+^ storage principle and the cycle stability.^209^ Reproduced with permission from ref. 209. Copyright 2023, Wiley-VCH GmbH. (d) Schematic representation of the nanocellulose gel electrolyte fabrication.^210^ Reproduced with permission from ref. 210. Copyright 2022, Wiley-VCH GmbH.

To develop wearable battery systems, the initial step involves designing flexible electrodes with high mechanical strength that can meet practical requirements. Carbon fiber cloth, due to its high electronic conductivity and excellent mechanical flexibility, serves as an ideal substrate for supporting free-standing and binderless flexible electrodes. For example, Zhao *et al.*^207^ fabricated a self-supporting and binding-free flexible SIB anode by preparing a carbon fiber cloth supported hollow rutile type titanium dioxide cube array composite (Fig. 22a). The unique structure of the hollow cube, attributed to the self-assembled TiO_2_ nano-cube, facilitates electrolyte ion transport and provides a large contact area for charge storage, resulting in near “zero strain” properties during charging and discharging processes. The composite exhibits remarkable performance (103.3 mA h g^−1^ at 50C) and cycle stability (95.6% after 600 cycles). Furthermore, when assembled into flexible devices, it demonstrates the ability to bend up to 180° while maintaining normal operation. Niu *et al.*^208^ synthesized a bimetallic selenide heterojunction composite (CoSe_2_/FeSe_2−*x*_) with carbon encapsulation on waste carbon cloth (CCFSF), effectively regulating the selenium-vacancy content (Fig. 22b). Due to the strong interaction between carbon and Se-metal (Co/Fe) interfaces, electron transfer is significantly accelerated, preventing the accumulation and agglomeration of loaded nanoparticles. The outermost carbon coating not only facilitates fast electron mobility and high electronic conductivity but also inhibits volume changes during sodiation/de-sodiation processes, ensuring excellent structural stability. Moreover, the assembled quasi-solid-state SIBs exhibit remarkable flexibility and energy storage performance.

In addition, reduced graphene oxide can also serve as a flexible electrode material substrate. To enhance the diffusion kinetics of Na^+^ ions, Sun *et al.*^209^ prepared a heterojunction composite of Na_5_V_12_O_32_/rGO (Fig. 22c). Within this heterostructure, rGO not only acts as a flexible substrate but also undergoes chemical transformation through interfacial electronic interactions with V–O bonds of NVO, resulting in controllable V–O–C bonds at the interface. The interfacial synergy between excellent bonding properties and inherent stress fields at the heterogeneous interface simultaneously reduces Na^+^ diffusion resistance and promotes charge transfer. As a result, the composite exhibits excellent rate performance (100 mA h g^−1^ at 10C) and cycling stability (95.4% retention after 3000 cycles).

#### 8.1.2 Flexible electrolytes

The low conductivity of electrolytes and structural collapse caused by repeated ion embedding/de-embedding during charging and discharging pose challenges for flexible SIBs. To address this problem, Niederberger *et al.*^210^ developed a biopolymer-based gel electrolyte for SIBs (Fig. 22d). By incorporating cellulose nanocrystals (CNC) with a continuous 3D structure, the collapse of the electrolyte structure is prevented. Furthermore, crosslinking CNC with cellulose nanofibers (CNFs) significantly enhances the diffusion rate of Na^+^ ions, resulting in a high conductivity of up to 2.32 mS cm^−1^. Additionally, Na/Na symmetric batteries utilizing the CNC/CNF gel electrolytes demonstrate stable operation at current densities as high as 0.5 mA cm^−2^ for nearly 100 h.

To construct high-performance flexible SIBs, it remains a challenge to simultaneously achieve high electrical conductivity and good cycling stability while maintaining the high mechanical strength of carbon matrix composites as well as gel electrolytes. The rational design and optimization of the layered structure and interfacial properties of carbon-based electrode materials play a crucial role in this endeavour.

### 8.2 Potassium ion batteries

**Fig. 23:**
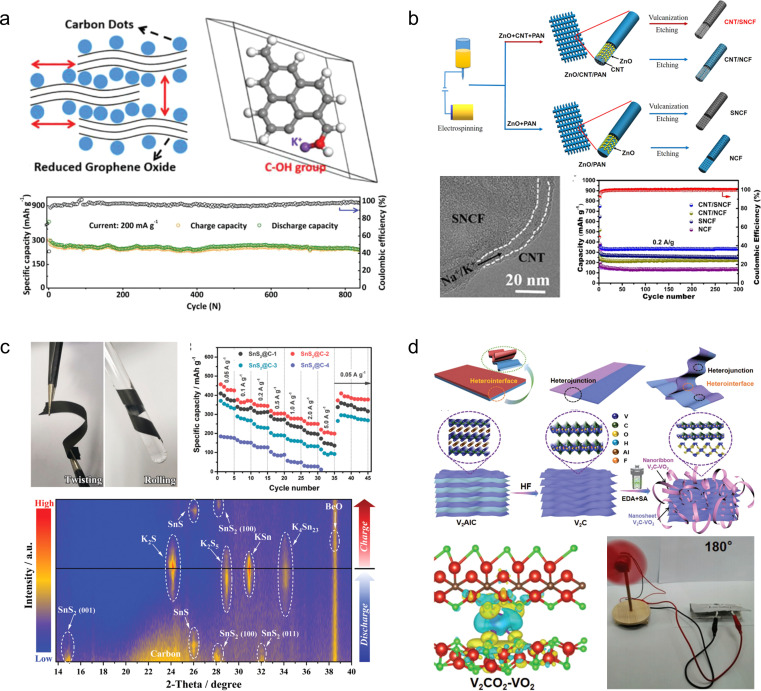
(a) Schematic diagram and long-term cycling performance at 200 mA h g^−1^ of CDs@rGO.^211^ (b) Schematic illustration for the synthesis, TEM, and long-term cycling performance of the CNT/SNCF composite.^212^ Reproduced with permission from ref. 212. Copyright 2021, American Chemical Society. (c) Physical demonstration, rate performance and *in situ* XRD patterns of SnS_2_@C.^213^ (d) Schematic illustration of the preparation process and practical application of the V_2_C–VO_2_ multi-heterostructure.^214^ Reproduced with permission from ref. 214. Copyright 2022, Wiley-VCH GmbH.

Electrode materials for flexible KIBs can be prepared by combining various carbon materials with electroactive substances. For instance, Lu *et al.*^211^ synthesized free-standing and flexible anode materials (CDs@rGO) for PIBs by incorporating carbon dots onto the surface of reduced graphene oxide (Fig. 23a). Due to the abundant defects and oxygen-containing functional groups provided by the carbon dots, CDs@rGO exhibited exceptional multiplicity performance and prolonged cycle life. However, as CDs@rGO is an unmodified pure carbon matrix composite, its capacity at 0.1 A g^−1^ is only 310 mA h g^−1^, which is unsatisfactory.

In order to achieve a higher specific capacity, Chen *et al.*^212^ proposed a unique multi-channeling carbon-based electrode material composed of N,S co-doped carbon nanotubes and carbon nanofibers (Fig. 23b). The co-doping of S,N co-doping not only significantly enhances the electrical conductivity of the composite material but also increases the interlayer spacing in the carbon material, effectively mitigating volume changes during cycling caused by K^+^ ion intercalation/deintercalation processes and thereby enhancing cycling stability. Based on this, Ci *et al.*^213^ further confined SnS_2_ within N,S co-doped carbon nanofibers as a flexible anode material for PIBs, achieving a remarkable reversible capacity of 457.4 mA h g^−1^ at 0.05 A g^−1^ (Fig. 23c). Through *in situ* XRD tests, the authors confirmed that the ultra-high reversible capacity can be attributed to the successive transformation alloying reactions and the enhanced capacitive behaviour exhibited by the SnS_2_ active material.

To achieve synergistic enhancement of capacity and mechanical properties, Xiao *et al.*^214^ fabricated a 3D multi-heterostructure composed of V_2_C–VO_2_ nanoribbons wrapped around nanosheets with abundant heterointerfaces and heterojunctions, which not only facilitates ion diffusion but also enhances the mechanical stability of the material (Fig. 23d). The authors further demonstrate that the V_2_C–VO_2_ multi-heterojunction can self-regulate the spin polarization density of its constituent monomers (V_2_C and VO_2_), thereby promoting K^+^ ion dynamics during charge/discharge cycling. As a result, V_2_C–VO_2_ exhibits satisfactory specific capacity (372 mA h g^−1^ at 0.2 A g^−1^) and excellent flexibility.

The LIBs, LSBs, SIBs and PIBs belong to the organic system of secondary batteries, which possess a wider voltage range and larger specific capacity than those employing hydrogel electrolytes; however, they also entail the risk of spontaneous combustion. To enhance the safety of flexible devices, numerous related strategies have been reported such as preparing non-flammable organic gel electrolytes and modifying fire-resistant coatings on electrode surfaces; nevertheless, these approaches inevitably result in certain electrochemical performance sacrifices for the devices. Furthermore, components utilized in organic system secondary batteries are poorly biocompatible and harmful to the human body; therefore, it is necessary to develop suitable encapsulation fabrics with skin-friendly properties to prevent direct contact between internal materials and human skin.

## 9. Conclusions and outlook

The advent of the smart electronics era necessitates the development of environmentally friendly, electrochemically superior, and lightweight flexible energy storage devices. However, the current performance of the developed flexible energy storage devices still falls short in meeting practical application demands. To advance wearable electronic device development, this review provides a comprehensive review on the research progress in various flexible energy storage systems. This includes novel design and preparation of flexible electrode materials, gel electrolytes, and diaphragms as well as interfacial engineering between different components.

The energy storage systems applied to wearable electronic devices in this review are categorized into two groups: water-based systems and organic-based systems. Water-based systems include SCs, ZIBs, and metal–air batteries, while organic-based systems consist of LIBs, LSBs, SIBs, and PIBs. Considering the direct contact between wearable electronic devices and human skin, safety is the primary concern. From a safety perspective, energy storage systems utilizing water gel electrolytes are safer than those using organic gel electrolytes. Moreover, hydrogels are more environmentally friendly and cost-effective compared to organic gels. However, these aqueous electrochemical energy storage devices have their own advantages and disadvantages in terms of performance: SCs offer fast charging and discharging but lack sufficient endurance; ZIBs exhibit higher energy storage efficiency but suffer from poor cycle life due to zinc dendrite growth, which is a defect that becomes amplified in flexible ZIBs; metal–air batteries exhibit satisfactory energy density and power density, but because the system is in direct contact with air, there are effects on the long-term stability of the hydrogel electrolyte, such as extreme temperatures leading to evaporation/freezing of water and side reactions between the electrolyte and oxygen in the air. On the other hand, despite the inadequate safety of organic energy storage devices, numerous strategies have been proposed to address this issue, including incorporating flame retardant additives into the system and utilizing non-combustible battery housings. Due to their utilization of organic gel electrolytes, these secondary batteries can circumvent hydrogen evolution and oxygen evolution reactions, thereby exhibiting a wide active voltage range and high energy density. While LIBs and LSBs encounter escalating costs, efforts are currently being made to substitute them with SIBs/PIBs; however, this comes at the expense of battery capacity.

Extensive research has been dedicated to employing diverse advanced material synthesis strategies for developing carbon-based/polymer-based composites with both mechanical and electrochemical properties. Nonetheless, there exist several significant bottlenecks impeding the commercialization of flexible energy storage devices:

(1) The energy storage performance of most flexible energy storage devices developed so far falls significantly short compared to that of non-flexible energy storage devices in similar systems, including cycle life, energy density, and power density. The primary reasons for this disparity are as follows: firstly, the preparation strategy for flexible electrode materials typically involves composite flexible substrates with active materials. However, most of these substrates exhibit low specific capacity and mass loading of active materials. Therefore, it is critical to develop flexible substrates with high specific surface area and exceptional electrochemical properties or design substrate-free flexible electrode materials. Secondly, the active material and the flexible substrate are connected by an unstable binder, serving as a mass transfer bridge, which results in a reduced power density. Additionally, there exists a potential risk of active material shedding caused by volumetric variations during the charge and discharge cycles. Furthermore, material deformation exacerbates the existing challenges faced by various energy storage systems such as Zn dendrite growth in ZIBs, the polysulfide-induced “shuttle effect” in LSBs, and electrolyte volatilization in ZABs, leading to degradation in cycle life.

(2) Currently, the research focus in the field of flexible energy storage devices primarily lies in the development of novel electrode materials, often overlooking other crucial components such as electrolytes, separators, and current collectors. However, it is important to recognize that these components do not operate independently; any performance mismatch among them may pose potential safety hazards during practical device utilization. Therefore, further advancements are required for non-electrode components of energy storage devices. Moreover, the concept of “all-in-one” energy storage devices holds great promise as it enables seamless integration between individual components.

(3) The majority of flexible energy storage devices are currently limited to laboratory production and have not yet been commercialized. To achieve cost-effective and large-scale fabrication of these devices, it is imperative to investigate simplified and controllable device integration technologies. Furthermore, to meet the practical requirements of wearable electronic devices, energy storage devices must be seamlessly integrated into flexible, breathable textiles. Therefore, efficient weaving techniques need to be developed as a replacement for manual laboratory weaving. Furthermore, the utilization of 3D printing technology enables the fabrication of flexible electrode materials and their integration into electronic device systems with intricate geometries tailored to specific requirements, thereby minimizing raw material wastage. In recent years, numerous studies have reported the application of 3D printing for electrochemical energy storage and conversion electrodes/devices due to its rapid prototyping capabilities and cost-effectiveness. However, ensuring both mechanical stability and device capacity remains a pressing challenge as the feedstock used in 3D printing often incorporates non-conductive substrates (*e.g.*, acrylonitrile butadiene styrene and polylactic acid), which can adversely impact device performance.

(4) With the increasing demand for wearable/portable electronics, the development of flexible energy storage devices has been progressively advancing towards versatility, intelligence, and scalability. However, most of the current flexible carbon-based composites possess a singular functionality and are limited to specific application scenarios (compression, tensile strain, and bending). Consequently, integrating multiple functionalities into a unified carbon matrix composite remains a significant challenge. Furthermore, exploring novel conductive polymer gel electrode materials could offer potential solutions. Recent efforts have focused on developing self-repairing, extreme temperature-resistant, and fatigue-resistant gel electrodes that hold promise for future wearable electronic devices.

(5) Regarding the safety of wearable electronics, apart from addressing issues such as electrolyte leakage and mechanical damage-induced leakage, there is also a growing focus on the comfort and biocompatibility aspects. Skin-friendly touch panels are essential for electronic devices and wearable electronics to prevent skin rashes or itching caused by prolonged usage. However, achieving touch interaction panels that are truly compatible with human skin remains a challenge in current designs of wearable electronics.

In the past decade, the interdisciplinary field of flexible electronics has experienced significant growth by surmounting the limitations associated with rigidity. It is anticipated that this domain will find extensive applications across various facets of human life, encompassing wearable sensors for monitoring human behavioral patterns, miniature mobile power sources with flexibility for portable exoskeletons, and implantable electronic devices for minimally invasive surgeries or diagnostic medical imaging in pathology. The convergence of flexible sensors and display technologies holds immense potential to facilitate the advancement of smart home and smart city planning. The capabilities offered by flexible sensors and Internet of Things devices could empower robots to play a pivotal role in disaster response and agricultural monitoring, as well as enabling real-time road traffic condition surveillance. Moreover, the development of flexible energy storage devices not only drives technological progress on Earth but also supports space exploration within the aerospace industry, ultimately enabling humanity's venture into cosmic realms.

## Data availability

The authors confirm that the data supporting the findings of this study are available within the article [and/or] its supplementary materials.

## Author contributions

All authors contributed to the writing and editing of this review article.

## Conflicts of interest

There are no conflicts to declare.
